# Two redundant transcription factor binding sites in a single enhancer are essential for mammalian sex determination

**DOI:** 10.1093/nar/gkae178

**Published:** 2024-03-18

**Authors:** Meshi Ridnik, Elisheva Abberbock, Veronica Alipov, Shelly Ziv Lhermann, Shoham Kaufman, Maor Lubman, Francis Poulat, Nitzan Gonen

**Affiliations:** The Mina and Everard Goodman Faculty of Life Sciences and the Institute of Nanotechnology and Advanced Materials, Bar-Ilan University, Ramat Gan 5290002, Israel; The Mina and Everard Goodman Faculty of Life Sciences and the Institute of Nanotechnology and Advanced Materials, Bar-Ilan University, Ramat Gan 5290002, Israel; The Mina and Everard Goodman Faculty of Life Sciences and the Institute of Nanotechnology and Advanced Materials, Bar-Ilan University, Ramat Gan 5290002, Israel; The Mina and Everard Goodman Faculty of Life Sciences and the Institute of Nanotechnology and Advanced Materials, Bar-Ilan University, Ramat Gan 5290002, Israel; The Mina and Everard Goodman Faculty of Life Sciences and the Institute of Nanotechnology and Advanced Materials, Bar-Ilan University, Ramat Gan 5290002, Israel; The Mina and Everard Goodman Faculty of Life Sciences and the Institute of Nanotechnology and Advanced Materials, Bar-Ilan University, Ramat Gan 5290002, Israel; Group “Development and Pathology of the Gonad”. Department of Genetics, Cell Biology and Development, Institute of Human Genetics, CNRS-University of Montpellier UMR9002, Montpellier, France; The Mina and Everard Goodman Faculty of Life Sciences and the Institute of Nanotechnology and Advanced Materials, Bar-Ilan University, Ramat Gan 5290002, Israel

## Abstract

Male development in mammals depends on the activity of the two SOX gene: *Sry* and *Sox9*, in the embryonic testis. As deletion of Enhancer 13 (Enh13) of the *Sox9* gene results in XY male-to-female sex reversal, we explored the critical elements necessary for its function and hence, for testis and male development. Here, we demonstrate that while microdeletions of individual transcription factor binding sites (TFBS) in Enh13 lead to normal testicular development, combined microdeletions of just two SRY/SOX binding motifs can alone fully abolish Enh13 activity leading to XY male-to-female sex reversal. This suggests that for proper male development to occur, these few nucleotides of non-coding DNA must be intact. Interestingly, we show that depending on the nature of these TFBS mutations, dramatically different phenotypic outcomes can occur, providing a molecular explanation for the distinct clinical outcomes observed in patients harboring different variants in the same enhancer.

## Graphical Abstract

**Graphical Abstract:**
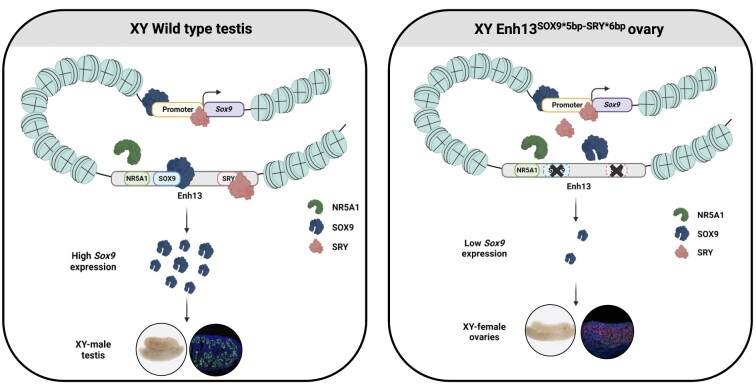

## Introduction

Genome-wide association studies (GWAS) indicate that the majority of genetic variants associated with human complex diseases map to non-coding parts of the genome which are far from gene bodies and promoters, probably in enhancers (1–3). Furthermore, while many variants are identified, it remains a major challenge to assign clinical causality to these variants (1).

Disorders of Sex Development (DSD) are defined as a group of congenital conditions in which the chromosomal, gonadal, or anatomical sex is atypical. DSDs affect 1:4000 newborns and are considered common birth defects in humans (4–7). Interestingly, the genetic manifestation of DSDs is an excellent example to the above stated as >50% of 46,XY DSD and ∼70% 46,XX DSD individuals fail to receive genetic diagnosis after Whole Exome Sequencing (WES) (4–6). This suggests that many DSD-causing variants fall within the non-coding parts of the genome and may overlap with regulatory elements.

The process of sex determination relies on two very delicate, antagonistic, genetic programs that determine the developmental path of an initially bipotential gonad into either a testicular or ovarian path (8–11). Testis differentiation is initiated by the activity of the Y chromosome-encoded gene *Sry* (sex determining region Y) which starts to be expressed in the supporting cell precursors at embryonic day (E)10.5 (12–14). SRY, together with NR5A1/SF1 activate the expression of *Sox9* (15–17). Once *Sox9* is expressed and reaches a critical threshold, it is considered the major pro-testicular factor responsible for the activation of thousands of target genes needed for testis development while opposing the ovarian developmental path (14,18). Indeed, *Sox9*/ *SOX9* loss-of-function in mouse and human leads to XY female development (14,19–22) while gain-of-function leads to XX male development (23,24). Several positive feedback loops exist to maintain high level of *Sox9* expression in the testis throughout life, including SOX9 regulating its own expression (8–11).

We have previously identified Enhancer 13 (Enh13) as a potent enhancer of the *Sox9* gene in the embryonic testis (15). Enh13 is a 557 bp long enhancer located 565 kb upstream of the *Sox9* transcription start site. Despite being one of several functional enhancers located upstream of *Sox9*, deletion of Enh13 in both mouse and human leads to XY male-to-female sex reversal (15,25,26). Furthermore, duplication of Enh13 results in XX female-to-male sex reversal in humans, possibly via overactivation of the *SOX9* gene (25,27). Enh13 was shown to be bound *in vivo* by both SRY and SOX9 itself (15).

Here, we explored the critical elements that control the activity of Enh13 and mediate male development. More specifically, we asked whether mutation in transcription factor binding sites (TFBS) within Enh13 can mediate the same phenotype as the deletion of the entire enhancer. To that aim, we generated mice carrying microdeletions in the NR5A1/SOX9/SRY TFBS of Enh13, or combinations of these microdeletions, and analysed their chromosomal sex and phenotype. We show that individual microdeletions in TFBS do not lead to XY sex reversal and while some affect gene expression, others do not. A deletion of 204 bp removing the SOX9 and SRY BS, as well as the sequence between them, induces XY sex reversal. Remarkably, combined microdeletions only in the SOX9 and SRY BS lead to complete XY male-to-female sex reversal showing that SOX binding motifs are crucial for Enh13 activity and therefore for male development.

## Materials and methods

### Animal ethics statement

All animals were maintained with appropriate husbandry according to Bar Ilan University ethics protocols 11-02-2020 and 61-11-2020. All mice strains were generated and maintained on a C57BL/6J genetic background. Primers used for genotyping are listed in Supplementary Table S1. At least ten different animals of each genotype from all mouse strains were analysed.

### Cloning of luciferase vectors

The following expression plasmids for luciferase assays were used: pCMV3-hWT1-KTS-C-HA (SINO BIOLOGICAL, Ref: HG12282-CY), pcDNA3-Flag-hSOX9 (A gift from Gerd Scherer to FP, described in (28)), pcDNA3-2X-MYC-hSF1, and pcDNA3-mSRY-4X-Myc. The open reading frames of the human NR5A1 and mouse SRY were cloned into pcDNA3 expression vector with an N-terminal 2× Myc tag for NR5A1 and C-terminal 4× Myc tag for the mouse SRY. Empty pcDNA3 vector was used as control.

To clone DNA inserts for luciferase plasmids, gDNA was extracted from earpieces of XY Enh13 WT and XY Enh13^SOX9*5bp-SRY*6bp-/−^ mice using DNeasy Blood & Tissue kit (Qiagen, 69504). Enh13 was amplified along with the addition of restriction enzymes BglII and HindIII sites using 2× Phusion (NEB, M0531L). Ogawa Enh13 sequence was synthesized along with restriction enzyme BglII and HindIII sites into the pUC57 plasmid by Syntezza Bioscience Israel. DNA inserts were digested using BglII and HindIII enzymes (Thermo, K1991) and ligated into the pGL4.26 plasmid (Promega) between the BglII and HindIII sites in the multiple cloning site using DNA ligation kit (Takara, 6022). Sanger sequencing was used to validate each ligation product. Primers used for the cloning can be found in Supplementary Table S1.

### Design and preparation of sgRNAs mRNA

Single guide RNAs (sgRNAs) were designed against each TFBS, using IDT CRISPR/Cas9 design tool (https://eu.idtdna.com). sgRNAs were chosen based on their proximity to the core of the TFBS with the assumption that the Cas9 cuts 3–4 bp upstream of the PAM site.

For creating crRNA-tracrRNA duplex, crRNA of all guides (sgRNAs sequences are listed in Supplementary Table S2) and tracrRNA (IDT, 224893246) were resuspended into final concertation of 200 μM using Nuclease Free Duplex Buffer (IDT, 11-01-03-01). crRNA and tracrRNA were mixed in 1:1 ratio, to a final complex concentration of 100 μM. To anneal the complex, the reaction was incubated at 95°C for 5 min, cooled to room temperature, and stored at –20°C for up to 2 weeks or until used.

CRISPR/Cas9 ribonucleoproteins (RNP) assembly was performed just prior to electroporation. RNP Mix was prepared within Opti-MEM (Thermo Fisher Scientific, 31985-062) and contained 1.2 μM Cas9 Nuclease V3 (IDT, 1081059) and 6 μM sgRNA. When two RNPs were electroporated, the amount of each sgRNA was reduced to 3 μM. RNP Mix was incubated at room temperature for 10 min and then placed on ice until used for zygote electroporation.

### Zygote harvesting and electroporation

For zygote harvesting, 4–7 weeks old C57BL/6J donor female mice were super-ovulated by administration of 5 IU of PMSG (Pregnant Mare Serum Gonadotropin; ProSpec, hor-272) using intraperitoneal (i.p.) injection. 48–50 h following PMSG injection, females were injected with 5 IU of hCG (Human chorionic gonadotropin; Sigma Aldrich CG10). Subsequently, super-ovulated females were mated with C57BL/6J adult males in 1:1 ratio and checked for the presence of vaginal plug (VP) the morning after mating. Female mice displaying VP were sacrificed via cervical dislocation for oviduct dissection and zygote isolation.

Oviducts were dissected, and ampulla nicked to release zygotes associated with surrounding cumulus cells into a M2 medium (Sigma Aldrich, M7167) containing 300 μg/ml hyaluronidase (Sigma Aldrich, H4272). Zygotes were picked using a mouth pipette and transferred to a plate containing fresh 2 ml M2 and subsequently passed through several M2 washes to remove cumulus cells. Next, zygotes were moved to a plate with KSOM medium (Mercury, MR-106-D). Zygotes were kept in KSOM medium in a flat-bed CO_2_ incubator (5.3% CO_2_, 5% O_2_ at 37°C) (Esco, Miri 2070047) for 30 min. Zygotes with the presence of polar bodies and pronucleus were isolated and washed in M2 drops followed by three washes in Opti-MEM drops and then moved to the electrode chamber containing the RNP mix.

Electroporation was performed using NEPA21 electroporator (NepaGene) in repeated pulses to first damage the zona pellucida and then promote the intracellular entry of RNPs. We used either large glass plate (CUY505P5 electrode with 5 mm gap) with 50 μl of RNP complex and 20–150 embryos or a small glass plate (CUY501P1 electrode with 1 mm gap) with 5 μl of RNP complex and 5–50 embryos. Electroporation parameters were as such: the poring pulse in the large glass plate was set to voltage: 225 V, pulse width: 1 ms, pulse interval: 50 ms and number of pulses: 4. The transfer pulse were set to voltage: 20 V, pulse width: 50 ms, pulse interval: 50 ms, and number of pulses: 5. For the small glass plate the poring pulse was set to voltage: 40 V, pulse width: 3.5 ms, pulse interval: 50 ms, and number of pulses: 4. The transfer pulse were set to voltage: 5 V, pulse width: 50 ms, pulse interval: 50 ms, and number of pulses: 5. Impedance was measured before and after embryo addition as stated by the manufacturer instructions. Following electroporation, zygotes were washed with M2 and KSOM drops and incubated in a KSOM drop overnight at CO_2_ incubator (5.3% CO_2_, 5% O_2_ at 37 °C). Finally, embryos that have developed to the 2-cell stage were surgically transferred into oviducts of pseudo-pregnant CD1 recipient females.

### gDNA isolation and genotyping genetically altered mice

Genomic DNA (gDNA) was extracted from tail tissue of embryos or ear punch tissue of adult animals. gDNA isolation from adult earpiece included 15 min incubation at 95°C with lysis buffer composed of 10 mM NaOH, 0.1 mM EDTA pH 8 followed by the addition of 40 mM Tris–HCl pH 5. For embryo samples, the PCRBIO rapid extract lysis kit was used (PCRBIO, PB15.11-S). Founder mice for the targeted mutation in the NR5A1, SRY or SOX9 binding sites within Enh13 were first analysed using Sanger sequencing of a 616 bp fragment containing the entire Enh13 (all genotyping primers are listed in Supplementary Table S1). Once a founder was identified that carries a desired mutation, it was bred to a C57BL/6J mice to get germline transmission. The F1 pups were also genotyped by Sanger sequencing after which a specific PCR reaction was designed based on the specific mutation (all genotyping primers are listed in Supplementary Table S1). Males and females F1 heterozygous mice for the same mutation were used to establish a colony of this specific mutation.

Two independent stable mouse strains carrying different size microdeletion/ larger deletions were established. All phenotypes were 100% penetrant and all XY Enh13^SOX9-SRY^**^Δ^**^204bp^, XY Enh13^SOX9-SRY^**^Δ^**^210bp^ and XY Enh13^SOX9*5bp^**^-^**^SRY*6bp^ embryos and mice were fully sex reversed.

For Sanger sequencing, PCR products were purified using universal DNA purification kit (TIANGENE, TI-DP214-03) or by EPPiC Fast Kit (A&A biotechnologies, 1021-500F) according to the manufacturer's instructions. INDEL (insertion-deletion) mutation analysis on the founder mice and F1 pups was done using the online TIDE tools software (https://tide.nki.nl), Synthego ICE Analysis (https://ice.synthego.com) and DECODR (https://decodr.org/).

For analysing deletions, Sanger sequencing was done on the deleted allele that was cut and purified from a 2% agarose gel (Invitrogen, 16500) using the QIAquick gel extracrtion kit (Qiagen, 28704) according to the manufacturer's instructions. Deletions were analysed at both founders and F1 mice. As the online INDEL tools are not very accurate for analysing two sequential mutations on the same allele, TA cloning (Promega pGEM®-T, A3600) was performed to accurately analyze the combined SRY and SOX9 BS mutations (Enh13^SOX9*SRY*^). Purified PCR product of the 616 bp Enh13 was ligated into the pGEM®-T vector. The ligated plasmid (pGEM®-T-Enh13) was transformed to competent *E. coli* bacteria (NEB, C3019) and incubated in Super Optimal broth (SOC) medium (Bioprep, SOCM-250ML) for 1 hour at 37°C. The transformed bacteria were seeded on the LB agar medium containing 100 μg/mL ampicillin (Formedium, AMP-5G) and incubated at 37°C for 18 h. Several single colonies were picked for DNA isolation using NucleoSpin Plasmid EasyPure kit (Macherey-Nagel, 740727). DNA was Sanger sequenced and analyzed using BLAST (https://blast.ncbi.nlm.nih.gov).

All PCR reactions were performed using 2× Red PCR Master Mix polymerase (PCR-BIO, PB10.23-10) according to the manufacturer's instructions. All mice and embryos were also genotyped for the chromosomal sex (all genotyping primers are listed in Supplementary Table S1).

### Time mating, gonad harvesting, preparation and imaging

Embryos and animals carrying the desired mutations were produced by crossing heterozygotes mice. Embryos were collected after time mating at embryonic day E13.5, where day 0.5 was determined by the presence of VP.

All bright field images of gonads were taken using the Nikon Eclipse Ts2R microscope with an exposure time of 10ms and analysed using the NIS-Elements D software.

For immunostaining, gonads from E13.5 embryos and 6 week-old postnatal mice were harvested and fixed overnight in 4% paraformaldehyde (Sigma Aldrich, P6148) in phosphate-buffered saline (PBS) at 4°C, washed three times with PBST (PBS with 0.1% Triton (Sigma Aldrich, 9002-93-1) at room temperature, incubated with 20% sucrose (Fisher BioReagents, BP220-1) overnight at 4°C and then embedded in OCT (Leica, 14020108926) and stored at -80°C until further use.

For RNA analysis of E13.5 embryos, paired gonad-mesonephros complexes were dissected out, the gonad was separated from the mesonephros, and a pair of gonads were snap frozen and stored in –80°C until further use for RNA isolation and gene expression analysis by qRT-PCR.

### Immunofluorescence staining

Immunofluorescence staining was performed on 10 μm-thick sagittal cryostat sections (Leica, CM3050-S). Antigen retrieval was performed for embryonic samples with DAKO (Target retrieval solution, Agilent, S1699) at 65°C for 30 min. Samples were then blocked in PBST containing 10% donkey serum (Sigma Aldrich, D9663) for 1 h and incubated with primary antibodies (diluted in PBST containing 1% donkey serum) overnight at 4ºC (All primary and secondary antibodies as well as dyes used are listed in Supplementary Table S3). Following three PBST washes, secondary antibodies were added for 1 h at room temperature (RT). Slides were then washed, dried and mounted (Polysciences 18606-20). All immunofluorescence slides were also stained with 4′,6-diamidino-2-phenylindole (DAPI; Invitrogen, D1306) to visualize nuclear DNA. Images were obtained with a Leica Microsystems SP8 confocal microscope.

### Fertility tests

For fertility tests, 6 week-old females (XX wild type or XY Enh13^SOX9*5bp^**^-^**^SRY*6bp−/−^) were single caged with a 6 week-old C57BL/6J male for a total period of 6 months. During this time period, the number of litters born as well as number of pups born per litter were recorded and documented.

### RNA isolation, cDNA preparation, and quantitative real-time polymerase chain reaction (qRT-PCR)

Total RNA was extracted from 6 week-old gonads using Qiagen RNeasy Plus mini kit (Qiagen, 74134) and from E13.5 gonad pairs using Qiagen RNeasy Plus micro kit (Qiagen, 74034). RNA yield was quantified with a NanoDrop spectrophotometer. 1μg of RNA was taken from 6 week-old gonads and 500 ng RNA from E13.5 gonads for cDNA preparation using the SuperScript™ III Reverse Transcriptase according to the manufacturer's instructions (Thermo Scientific, 18080085).

qRT-PCR reactions were performed in duplicate using PowerSYBR Green PCR master mix (Thermo Scientific, AB-4367659) with 140 nM each of forward and reverse primers (Supplementary Table S4) and analyzed on the QuantStudio 1 Real-Time PCR System (Thermo Scientific). Analysis was done using Comparative CT (^ΔΔCT^) technique and expression is relative to the house keeping gene-*Hprt*. Statistical analyses were carried out using Prism 9 software (GraphPad) using one-way ANOVA test followed by Dunnett's post-test. *P* value <0.05 was considered as statistically significant. The number of gonads analyzed at each stage is depicted in the graphs.

### Electromobility shift assay (EMSA)

Expression Plasmids with T7 promoter were previously described for pcDNA3 mouse SRY (29), pcDNA3 human SOX9 and pcDNA3 human NR5A1 (30). Plasmid pCMV3 human WT1-KTS was obtained from Sino Biological (HG182-CY). Proteins were produced using the TNT^TM^ coupled translation system (Promega) using T7 RNA polymerase according to the manufacturer's instructions. Negative control was programed with pcDNA3 empty vector.

Probes were annealed by mixing 10 μg of each complementary oligonucleotides and heating at 80°C for three minutes in 20 mM Hepes pH 7.9, 50 mM NaCl, 6 mM MgCl_2_, slow-cooled to 50°C for 10 min and cooled to room-temperature. After annealing the DNA duplexes were de-salted through Sephadex G-50 spin column (GE healthcare, 27-5330-01). For radioactive labelling, a 5′ overhang G at both end of probes was used to fill in with α[^32^P]-dCTP (Amersham) using Superscript IV reverse transcriptase (Invitrogen). 100 ng of hybridized probes were incubated with 10 μCi of dCTP and 1 μl of Superscript IV for 1 h at 37°C in 1× RT buffer from manufacturer. Unincorporated nucleotides were removed through Sephadex G-50 spin column.

DNA binding assay were performed in a final volume of 20 μl with 10 mM Tris pH8, 100 mM KCl, 5 mM MgCl_2_, 10 mM ZnSO_4_, 1 mM Spermidine, 0.075% triton X100, 1 μg BSA, 1 mM DTT, 10% glycerol and 4 μl of *in vitro* translation mix. As non-specific competitors, for SOX9 or SRY, 0.1 μg of poly dG-dC and for NR5A1 or WT1, 0.1 μg of poly dI-dC were added. 1ng of radio-labeled probe was added, binding reactions were performed on ice for 20 minutes and complexes were resolved on a 5% native acrylamide (37.5:1) gel in 0.5× TB at 4°C, then dried and revealed using Molecular Dynamics PhosphoImager. Quantifications were performed using ImageQuant software. Statistics were performed using GraphPad Prism 9 with Ordinary one-way ANOVA with Tukey's multiple comparisons test (Supplementary Table S7).

#### Sequences of oligos for probes

NR5A1 BS Enh13 Wt fw: gCTGAGGAATTAGAAGGCCAGGTTGGC

NR5A1 BS Enh13 Wt rev: gGCCAACCTGGCCTTCTAATTCCTCAG

NR5A1 BS Ridnik et al fw: gCTGAGGAATCAGGTTGGC

NR5A1 BS Ridnik et al rev: gGCCAACCTGATTCCTCAG

SOX9 BS Enh13 Wt fw: gGGCTTGGGCAAGCAAACCACAACAATGGTCAGACTGATAA

SOX9 BS Enh13 Wt rev: gTTATCAGTCTGACCATTGTTGTGGTTTGCTTGCCCAAGCC

SOX9 BS Ridnik et al fw: gGGCTTGGGCAAGACTGATAA

SOX9 BS Ridnik et al rev: gTTATCAGTCTTGCCCAAGCC

SOX9 BS Ogawa et al fw: gGGCTTGGGCAAGCAAACCAGGGGGGGGGTCAGACTGATAA

SOX9 BS Ogawa et al rev: gTTATCAGTCTGACCCCCCCCCTGGTTTGCTTGCCCAAGCC

SRY BS Enh13 Wt fw: gGGTGTAGAGTCCACTTCTAAACAAACAGCTGAGGGG

SRY BS Enh13 Wt rev: gCCCCTCAGCTGTTTGTTTAGAAGTGGACTCTACACC

SRY BS Ridnik et al fw: gGGTGTAGAGTCCACAGCTGAGGGG

SRY BS Ridnik et al rev: gCCCCTCAGCTGTGGACTCTACACC

SRY BS Ogawa et al fw: gGGTGTAGAGTCCACTGGGGGGGGGACAGCTGAGGGG

SRY BS Ogawa et al rev: gCCCCTCAGCTGTCCCCCCCCCAGTGGACTCTACACC

WT1 BS fw: gGGCTTGGGCAAGCGGGGGAGGACAATGGTCAGACTGATAA

WT1 BS rev: gTTATCAGTCTGACCATTGTCCTCCCCCGCTTGCCCAAGCC

### Luciferase assay

All Luciferase assays were performed in HEK293T cells. 2.5 × 10^4^ cells were plated on 96-well plate 24 h prior to transfection. Cells were transfected with either empty or Enh13 WT/Enh13^SOX9*5bp^**^-^**^SRY*6bp^/Enh13 Ogawa pGL4.26 plasmids (100 ng) and expression plasmids for transcription factors (20 ng each) along with 1 ng of Renilla Luciferase pLR-TK vector (Promega). Transfection was performed using PolyJet transfection reagent (SignaGen, SL100688) at 3:1 polyJet : DNA ratio. Luciferase activity was measured after 24 h using the Dual-Glo® Luciferase Assay System according to the manufacturer's instructions (Promega, E2980).

To calculate relative Luciferase activity, Firefly Luciferase values were normalized to the Renilla Luciferase values and then to the average of the empty pcDNA3 vector. For Luciferase assays containing WT1, we normalized the Firefly luciferase values to that of the average of the empty pcDNA3 vector without normalizing first to Renilla as WT1 is known to affect Renilla activity (31). All experiments were performed in duplicate of technical repeats and were repeated at least three independent times. Statistical analyses were carried out using the Prism 9 software (GraphPad). Two-way ANOVA was performed to compare between samples followed by Tukey's/Dunnett's.

### Conservation analysis

Orthologous sequences between NR5A1 and SRY motifs of mouse En13 were obtained from assemblies of various mammalian genomes using LiftOver (https://genome.ucsc.edu/cgi-bin/hgLiftOver) and verified using BLAT from UCSC and Ensembl. Sequence where aligned using Muscle in SnapGene. Mouse (mm10), chr11:112217398–112217642. Rat (Rn7), chr10:97214983–97215227. Golden hamster (mesAur1.0), KB708147:9490541–9490779. Elephant (loxAfr3), scaffold_49:11080077–11080220. Dolphin (tuTru2), JH476380:163786–163930. Chimpanzee (panTro3) 17:70924554–70924783. Rabbit (oryCun2), 19:55204231–55204458. Horse (equCab3): chr11:9765148–9765290. Pig (susScr11), chr12:9209027–9209170. Human (hg38), chr17:71484768 –71484997.

## Results

### Mutations in individual TFBS do not cause sex reversal

**Figure 1.**
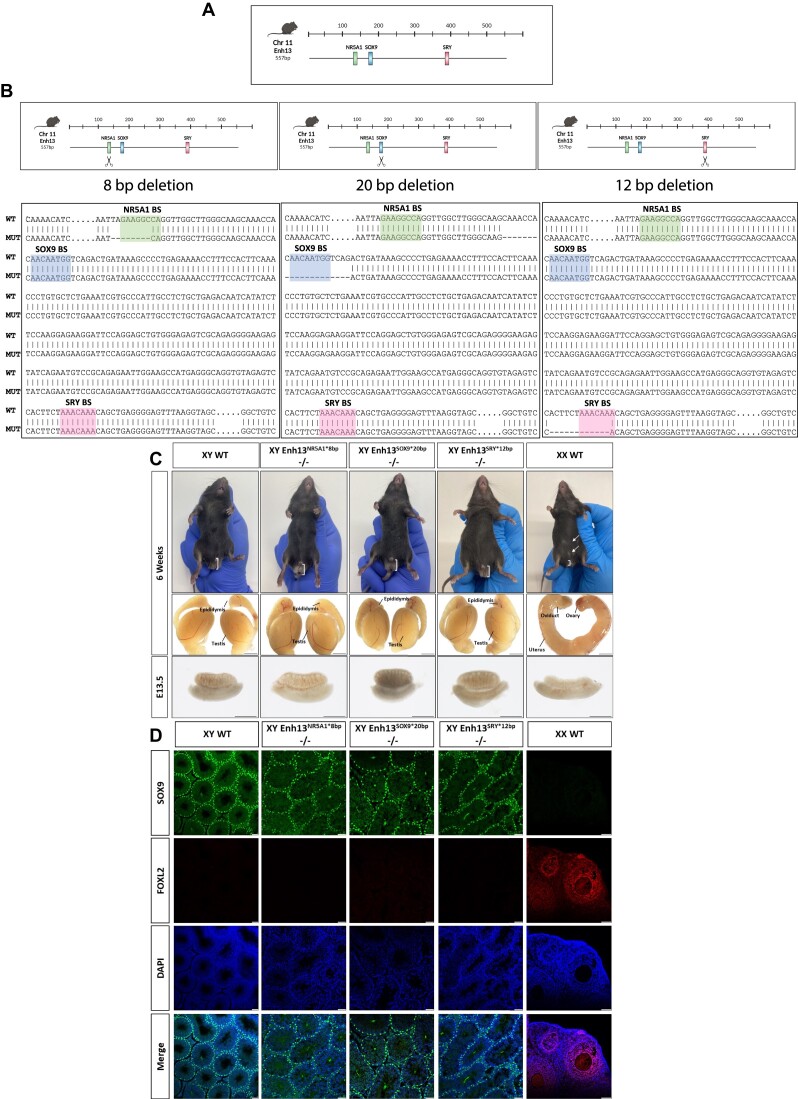
Mutations in individual TFBS of Enh13 do not cause sex reversal. (**A**) Schematic representation of the putative locations of the NR5A1 (green), SOX9 (blue) and SRY (pink) TFBS in Enh13 (557 bp) according to binding sites motif analysis tool (15). (**B**) Schematic representation of the CRISPR targeting approach and BLAST sequencing alignment between the WT Enh13 sequence (top) and homozygous mice mutated for Enh13 sequences (bottom) (NR5A1 BS- 8 bp deletion, SOX9 BS- 20 bp deletion, SRY BS- 12 bp deletion). (**C**) Bright field images of the external genitalia and gonads of 6 week-old mice and E13.5 embryos of WT male (XY) and female (XX), and XY homozygous mice of the different mutations in individual TFBS in Enh13 (NR5A1 BS- 8 bp deletion; Enh13^NR5A1*8bp^, SOX9 BS- 20 bp deletion; Enh13^SOX9*20bp^, SRY BS- 12 bp deletion; Enh13^SRY*12bp^). Scale bar represents 2000 μm for 6 week-old gonads and 500 μm for E13.5 gonads. (**D**) Immunostaining of 6-week-old gonads from WT XY and XX as well as XY homozygous mice of the different mutations in individual TFBS in Enh13 (Enh13^NR5A1*8bp^, Enh13^SOX9*20bp^, Enh13^SRY*12bp^). Gonads were stained for the Sertoli-marker SOX9 (green), granulosa-marker FOXL2 (red) and DAPI (blue). Scale bars represent 50 μm. WT- *Wild Type*

The mouse Enh13 contains several putative transcription factor binding motifs for NR5A1/SF1, SOX9, SRY and WT1 (Figure 1A and (15)). SRY and SOX9 have been previously shown to bind Enh13 *in vivo* using ChIP-qPCR at E11.5 or E13.5, respectively (15). As SRY and SOX9 are critical regulators of the testis path, and both are known to work with NR5A1/SF1 (11,17,30,32), we decided to focus on these three TFBS (Figure 1A). Indeed, conservation analysis among 10 mammalian species indicates that the NR5A1, SOX9 and SRY binding motifs are highly conserved (Supplementary Figure S1).

To examine if mutations in individuals TFBS within Enh13 can mediate the same phenotype as deleting the entire enhancer, CRISPR genome editing was used to generate mice containing random insertions/deletions (INDELs) in each of the three TFBS (Figure 1B and Supplementary Figures S2 and S3, termed Enh13^NR5A1*8bp^, Enh13^SOX9*20bp^ and Enh13^SRY*12bp^). Two independent mutated mouse strains, carrying different size microdeletions, were generated for each TFBS (Figure 1B, Supplementary Figure S2 and S3). Examination of external genitalia, as well as gonads at both adult and embryonic stages demonstrated that XY mice carrying heterozygous (Supplementary Figure S3) or homozygous (Figure 1C) microdeletions in NR5A1, SOX9 or SRY TFBS develop as *wild type* XY males. The same was true also for the second independent mutated strain for each of the three TFBS and they all presented as normal XY males (Supplementary Figure S2B-C). To further explore if the gonads show signs of sex reversal or ovotestis appearance, immunostaining analysis of the testis at embryonic (E13.5, Supplementary Figure S4) and adult (6 week-old, Figure 1D) stages was conducted and indicated that all XY mutated gonads are indistinguishable of *wild type* testis. Testis of homozygous XY Enh13^NR5A1*8bp^, Enh13^SOX9*20bp^ and Enh13^SRY*12bp^ presented with normal testis cords at embryonic stages and seminiferous tubules at adult stages which strongly expressed the Sertoli cell marker SOX9 while being devoid of the granulosa cell marker FOXL2 (Figure 1D, Supplementary Figure S4). All the above suggest that individual mutations in TFBS within Enh13 do not lead to the XY sex reversal phenotype observed upon deletion of the entire enhancer.

### Mutations in individual TFBS do not substantially alter gene expression

**Figure 2.**
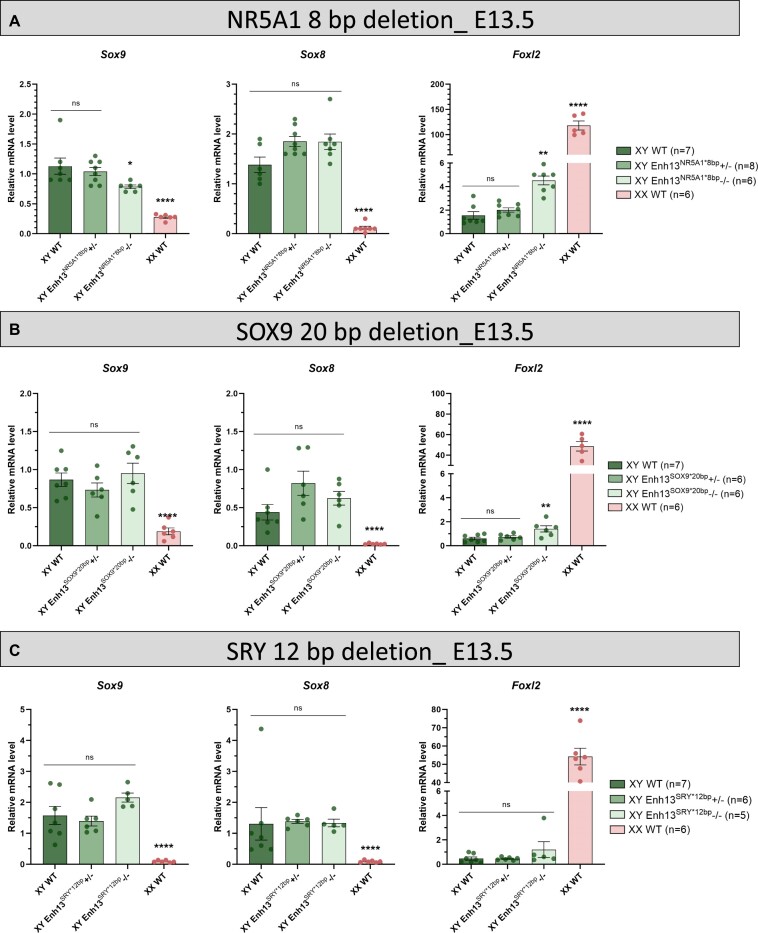
Gene expression analysis of mice carrying individual TFBS mutations in Enh13. Real-time quantitative PCR analysis of genes involved male (*Sox9* and *Sox8*) and in female (*Foxl2*) gonadal sex determination in E13.5 gonads of embryos carrying the different mutations in individual TFBS in Enh13 (Enh13^NR5A1*8bp^, Enh13^SOX9*20bp^, Enh13^SRY*12bp^). Data are presented as mean 2^−ΔΔCt^ values ± SEM, normalized to the housekeeping gene *Hprt*. Sample size indicated next to each genotype represents biological replicates, i.e. the number of individuals harvested. Statistical analysis was done using one-way ANOVA followed by Dennett's posttest. *P < 0.05, **P < 0.01, ***P < 0.001 and ****P < 0.0001, ns: not significant. WT: wild type.

The term ‘enhancer additivity’ was coined and refers to several enhancers working together to regulate the same gene, where each enhancer provides a set of percentage of the total transcription output produced (33–36). We speculated that perhaps additivity in TF also exist where each TFBS is responsible for a certain percentage of the total output of the enhancer activity (33). To explore this, quantitative PCR (qPCR) analysis was conducted on both embryonic (Figure 2) and adult (Supplementary Figure S5) gonads to examine the expression levels of *Sox9* itself, as well as *Sox8*, one of the first genes to be activated in the male cascade, and *Foxl2*, which is normally expressed in female granulosa cells. No significant change in gene expression was observed in heterozygous and homozygous gonads of each of the three TFBS mutated strains in adult gonads (Supplementary Figure S5). On the contrary, gonads from E13.5 XY embryos carrying a homozygous mutation in Enh13^NR5A1*8bp^ displayed a reduction of 30% in the expression levels of *Sox9 (*Figure 2A). This was accompanied by 3-fold increase in the expression levels of *Foxl2*. No change was observed with the expression of *Sox8* (Figure 2A). This may suggest that the NR5A1 transcription factor binding motif is responsible for 30% of the regulatory activity of Enh13 on the *Sox9* gene at embryonic stages.

Unlike the expression changes seen upon mutating the NR5A1 binding site, no major changes in expression were observed upon mutations in the SOX9 or SRY TFBS at XY embryonic gonads (Figure 2B, C). A slight activation in *Foxl2* expression was seen upon homozygous mutation in the SOX9 TFBS, yet no decrease in *Sox9* expression was evident (Figure 2B).

### Combined mutations in the SOX9 and SRY TFBS lead to XY sex reversal

**Figure 3.**
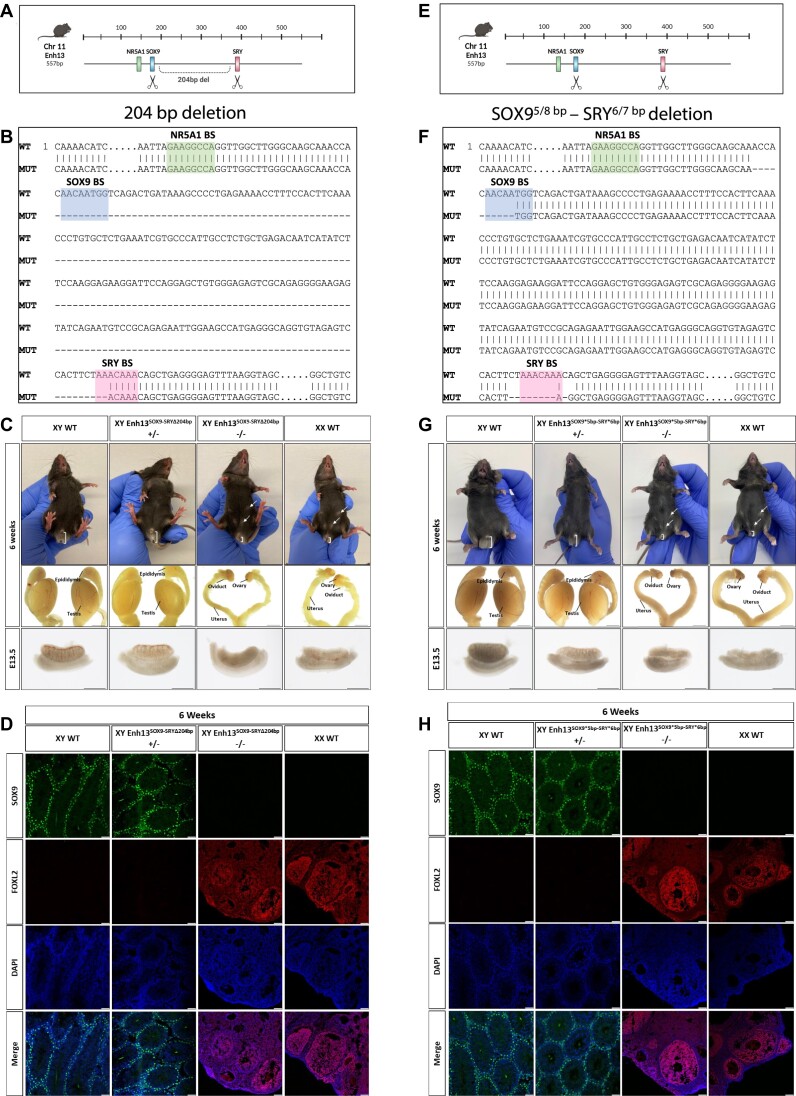
Combined mutations in the SOX9 and SRY TFBS of Enh13 lead to XY sex reversal. Characterization of mouse strain carrying a 204 bp deletion within Enh13 deleting the SOX9 and SRY TFBS and the sequence between them (XY Enh13^SOX9-SRY^**^Δ^**^204bp^) (A–D) and mouse strain carrying mutations only in the SOX9 and SRY TFBS (XY Enh13^SOX9*5bp^**^-^**^SRY*6bp^) (E–H). (A, E) Schematic representation of the CRISPR targeting approach in the mouse strains carrying deletion between the SOX9 and SRY TFBS (**A**) or mutations in both SOX9 and SRY TFBS (**E**). (B, F) BLAST sequencing alignment between the WT Enh13 sequence (top) and homozygous mice deleted (**B**) or mutated (**F**) Enh13 strains (bottom). (C, G) Bright field images of the external genitalia and gonads of 6 week-old mice and E13.5 embryos of WT male (XY) and female (XX) mice, and the XY heterozygous and homozygous mouse strains carrying deletion between the SOX9 and SRY TFBS (**C**) or mutations in both SOX9 and SRY TFBS (**H**). XY heterozygous mice present as male whereas XY homozygous mice present with complete male-to-female sex reversal. Scale bar represents 2000 μm for 6 week-old gonads and 500 μm for E13.5 gonads. (D, H) Immunostaining of 6 week-old gonads from WT XY and XX as well as XY heterozygous and homozygous mice with the deletion (**D**) or two TFBS mutations (H). Gonads were stained for Sertoli-marker SOX9 (green), granulosa-marker FOXL2 (red) and DAPI (blue). Scale bars represent 50 μm. WT- *Wild Type*

As mutations in the NR5A1 transcription factor binding motif lowered *Sox9* expression, yet mutations in either SOX9 or SRY TFBS had no major effect on gene expression, this prompted the hypothesis that the SOX9 and SRY TFBS are redundant to each other. Indeed, it has previously been shown that SOX9 and SRY can bind each other's binding sites quite efficiently and substitute each other, being close relatives of the SOX family proteins (37–40). Perhaps, upon mutation in the SOX9 binding site (BS), the two proteins can still bind via the SRY BS and when the SRY BS is mutated, they both operate via binding to the SOX9 BS. To experimentally explore this hypothesis, we established a mouse strain containing 204 bp deletion removing all of the SOX9 BS, half of the SRY BS and all the sequence between them, leaving the NR5A1 BS intact (Figure 3A, B, termed Enh13^SOX9-SRY^**^Δ^**^204bp^).

Analysis of external and internal genitalia at adult and embryonic stages indicated that XY mice carrying a homozygous 204 bp deletion are completely sex reversed presenting as females with ovaries, oviduct, uterus, and short anogenital distance, normally seen in females (Figure 3C). The same phenotype of complete XY male-to-female sex reversal was observed in XY homozygous mice from a second, independent strain carrying a 210 bp deletion removing most of the SOX9 and SRY BS and the sequence between them, leaving the NR5A1 BS intact (Supplementary Figure S6A, B, termed Enh13^SOX9-SRY^**^Δ^**^210bp^). All XY homozygous embryos and mice from these strains presented with 100% penetrance and complete sex reversal phenotype. Immunostaining analysis of gonads at embryonic (Supplementary Figure S7) and adult (Figure 3D) stages indicated that homozygous XY mice have normal-looking ovaries, expressing FOXL2 and are indistinguishable of XX mice, while heterozygous mice carrying the deletion are males with normal testis and expression of SOX9. This phenotype is identical to the phenotype observed upon deletion of the entire Enh13 (557 bp) (15).

Since removal of both the SOX9 and SRY BS using a 204 bp deletion led to full sex reversal, suggesting that the two BS are indeed redundant, we wanted to explore whether a more surgical removal of only these two TFBS will mediate the same phenotype. This will also verify that no critical elements are present in the ∼200 bp between the two sites. To that end, mice were generated containing microdeletions only in the SOX9 and SRY binding sites, leaving the NR5A1 BS and the rest of Enh13 intact (Figure 3E). These mice contained deletion of 5 out of 8 bp of the SOX9 BS and 6 out of 7 bp of the SRY BS (Figure 3F, termed Enh13^SOX9*5bp^**^-^**^SRY*6bp^). Quite remarkably, homozygous mice carrying microdeletions only in the SOX9 and SRY BS presented with complete XY male-to-female sex reversal and were indistinguishable of XX *wild type* females at both embryonic and adult stages (Figure 3G-H, Supplementary Figure S8). Gonadal analysis using immunostaining at both embryonic (Supplementary Figure S8) and adult (Figure 3H) stages indeed presented normal-looking ovaries in homozygous XY Enh13^SOX9*5bp^**^-^**^SRY*6bp^ mice while XY heterozygous mice had testes. All XY homozygous embryos and mice from this strain presented with 100% penetrance and complete sex reversal phenotype.

Next, we wanted to assess the fertility capabilities of the XY homozygous Enh13^SOX9*5bp^**^-^**^SRY*6bp^ female mice. To that aim, we performed fertility tests using either XX wild type C57BL/6J females or the XY homozygous C57BL/6J Enh13^SOX9*5bp^**^-^**^SRY*6bp^ females. We used 6 week-old females and bred them to 6 week-old males. Breeding cages were monitored for a period of 6 months and the number of litters born and well as number of pups were recorder. As can be seen in Supplementary Figure S9, the XY homozygous C57BL/6J Enh13^SOX9*5bp^**^-^**^SRY*6bp^ females are completely infertile, and no litters or pups were born during a period of 6 months.

### Nucleotide substitutions of TFBS can create unintended, novel binding sites

**Figure 4.**
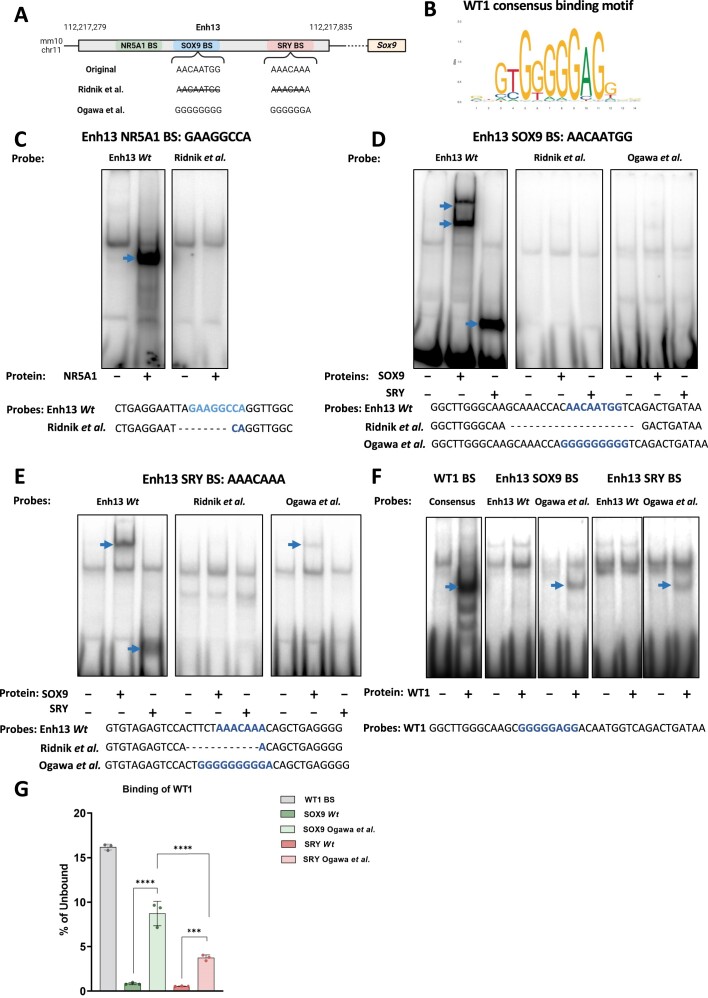
Electromobility shift assay (EMSA) analysis of the various mutations in TFBS in Enh13. (**A**) Schematic representation of the mouse Enh13 region and genomic coordinates (mm10) as well as the locations of the NR5A1 (green), SOX9 (blue) and SRY (pink) TFBS. The sequence of the SOX9 and SRY BS as they appear in Enh13 are presented and below them appears the microdeletions generated in this study (Ridnik *et al.*) and the Poly G sequences inserted by Ogawa *et al.*, aimed to destroy the SOX9 and SRY TFBS (41) (**B**) The consensus binding motif of the Wilms' tumor 1 (WT1) transcription factor. (**C**) EMSA using a probe containing the NR5A1 BS sequence of Enh13 (left) and a mutated probe containing an 8 bp deletion around the NR5A1 BS (right). Both probes were loaded in the absence or presence of NR5A1 protein. Blue arrow highlights the shifted probe due to binding in the presence of NR5A1 protein. (**D**) EMSA using a probe containing the SOX9 BS sequence of Enh13 (left) and two mutated probes containing either 20 bp deletion around the SOX9 BS (middle) or a Poly G sequence (right) (41). All three probes were loaded in the absence or presence of SOX9 or SRY protein. Blue arrows highlight the shifted probe due to binding in the presence of SOX9 or SRY proteins. (**E**) EMSA using a probe containing the SRY BS sequence of Enh13 (left) and two mutated probes containing either 12 bp deletion around the SRY BS (middle) or a Poly G sequence (right) (41). All three probes were loaded in the absence or presence of SOX9 or SRY protein. Blue arrows highlight the shifted probe due to binding in the presence of SOX9 or SRY proteins. (**F**) EMSA using probes containing either a WT1 consensus sequence (left), or two versions of probes for the Enh13 SOX9 BS (two middle gels) or the Enh13 SRY BS (two right gels). The first one is the *wild type* (*Wt*) Enh13 sequence, and the second one is a probe containing a Poly G sequence as used by Ogawa *et al.* (41). All five probes were loaded in the absence or presence of the WT1 protein. Blue arrows highlight the shifted probe due to binding in the presence of WT1 protein. For all experiments, negative control (–) correspond to lysate generated with empty expression vector. (**G**) Quantification of the binding capacity of WT1 protein to the five different probes. Quantification was done based on triplicate EMSA experiments (Supplementary Figure S8). Data presented as the percentage of bound probe out of the unbound probe. Error bars are the mean values ± SD. **P< 0.032; ***P< 0.0002; ****P< 0.0001. The sequences of all probes appear below the relevant EMSA gel. Blue highlights the TFBS, ‘–’ represents the microdeletions generated. Wt, wild type, WT1, Wilms' tumor 1.

Interestingly, a recent study also explored the functional elements of Enh13 (41). They created mice carrying either individual TFBS mutations in the SOX9 or SRY binding sites of Enh13 or mice carrying combined mutations in both the SOX9 and SRY binding sites. They did not use INDELs to mutate the binding sites but rather inserted a poly G sequence aiming to eliminate the binding capacity of the two TFs to their binding sites (Figure 4A). Strikingly, their phenotype was different than ours. They found that homozygous mice with individual poly G substitutions of either the SOX9 or SRY BS do not lead to any major gonadal phenotype, yet XY mice with homozygous poly G substitutions of both SOX9 and SRY BS are still males but present with small testis (41). This phenotype is different from the complete XY sex reversal observed upon homozygous microdeletions of the same TFBS on the same C57BL/6J genetic background (Figure 3G). Since the only difference between the two studies was that they introduced a poly G sequence while we introduced microdeletions in the TFBS, this prompted us to hypothesize that this poly G substitution may have created a novel, unintended TFBS for a TF that is present in the gonad, which mediated a partial rescue leading to different, and milder, gonadal phenotype. Interestingly, the Wilms’ Tumor 1 (WT1) protein binding site is very G rich (Figure 4B) and WT1 is known to be highly expressed at the bipotential gonad and then in Sertoli and granulosa cells (11,42–44). Knock out or mutations in WT1 can lead to both XY and XX gonadal dysgenesis in human and mouse (45–50). To examine this hypothesis, we performed electromobility shift assay (EMSA) using *in vitro* translated proteins, to explore the binding of NR5A1, SOX9, SRY and WT1 to the *wild type* sequences of Enh13 and the microdeletions/ poly G substituted sequences.

First, a probe containing the *wild type* sequence of the NR5A1 TFBS in Enh13 was used alongside a probe containing 8 bp microdeletion in the NR5A1 BS in Enh13 as present in our CRISPR mice (Figure 4C, Figure 1B, Supplementary Table S5). As evident in Figure 4C, we observed a strong complex of the *wild type* probe with the *in vitro* translated NR5A1 protein, not present with the 8 bp deleted probe that lack NR5A1 BS. This suggest that indeed the mice carrying 8 bp deletion in the NR5A1 BS of Enh13 are not able to bind NR5A1.

As SOX9 or SRY are predicted to bind the Enh13 SOX9 BS (37–39), three probes related to this region were generated covering either the *wild type* sequence of the SOX9 BS of Enh13, the 20 bp microdeletion spanning the SOX9 BS of Enh13 as present in our CRISPR mice (Figure 1B) or the poly G substitutions in the SOX9 BS of Enh13 as used in Ogawa et al., (41) (Figure 4A, Supplementary Table S5). As expected, the *wild type* sequence of Enh13 spanning the SOX9 BS is bound by either SOX9 or SRY (Figure 4D left panel). Interestingly, in presence of SOX9 two distinct complexes are observed in contrast with SRY, suggesting that SOX9 may bind DNA as a dimer, as already observed for SOX’s subgroup E (51). These findings support our hypothesis that both SOX9 and SRY are redundant in their ability to bind the SOX9 BS of Enh13. In contrast, no specific complexes were detected with the probe containing the 20 bp deletion as for the probe containing the Poly G sequence showing, in both cases, a complete loss of binding of SOX9 and SRY.

Similarly, we analysed three probes spanning the SRY BS in Enh13 and containing either the *wild type* SRY BS, the 12 bp microdeletion around the SRY BS as present in our CRISPR mice (Figure 1B) and the poly G substitutions in the SRY BS of Enh13 as used in Ogawa et al., (41) (Figure 4A, Supplementary Table S5). Again, the *wild type* Enh13 probe demonstrated binding by both SOX9 and SRY (Figure 4E) with a single complex, with an apparent weaker affinity when compared to the SOX9 BS (Figure 4D) suggesting that the SOX9 BS is the more potent BS out of the two binding sites. As expected, the 12 bp microdeletion of the SRY BS completely abolished the binding of both SOX9 and SRY (Figure 4E). Interestingly, the substitution of SRY BS by a poly G sequence induces a faint binding of SOX9, in contrast with SRY (Figure 4E).

Lastly, we wanted to examine whether a GC-rich motif binding transcription factor as WT1 might interact with the G-substituted motifs of SOX9 and SRY BS from Enh13 described by Ogawa *et al.* (41). As control, *in vitro*-translated WT1 binds strongly to its consensus binding motifs (Figure 4F, left panel). Interestingly, WT1 binds also, albeit less strongly, to the Poly G mutated probes of the SOX9 or SRY BS (Figure 4F). By contrast, WT1 displays no affinity for the *wild type* Enh13 probes. Quantification of the WT1 binding confirms a significant binding of WT1 to the Poly G sequences in the SOX9 and SRY BS compared to the *wild type* sequences of Enh13 (Figure 4G, Supplementary Figure S10, Supplementary Table S6-S7). These finding suggest that WT1 (or any other GC-rich motif binding transcription factors) could bind both the SOX9 BS and SRY BS when these are substituted with a poly G sequence.

Altogether, the EMSA experiments show that the predicted binding motifs are effectively bound by the corresponding transcription factor(s). Moreover, both SRY and SOX9 BS within Enh13 can bind WT1 when their sequences are replaced by G-rich motifs. This may confer partial rescue which results in a milder phenotype in mice when compared to the INDEL mutations inserted at the same SOX9 and SRY BS.

**Figure 5.**
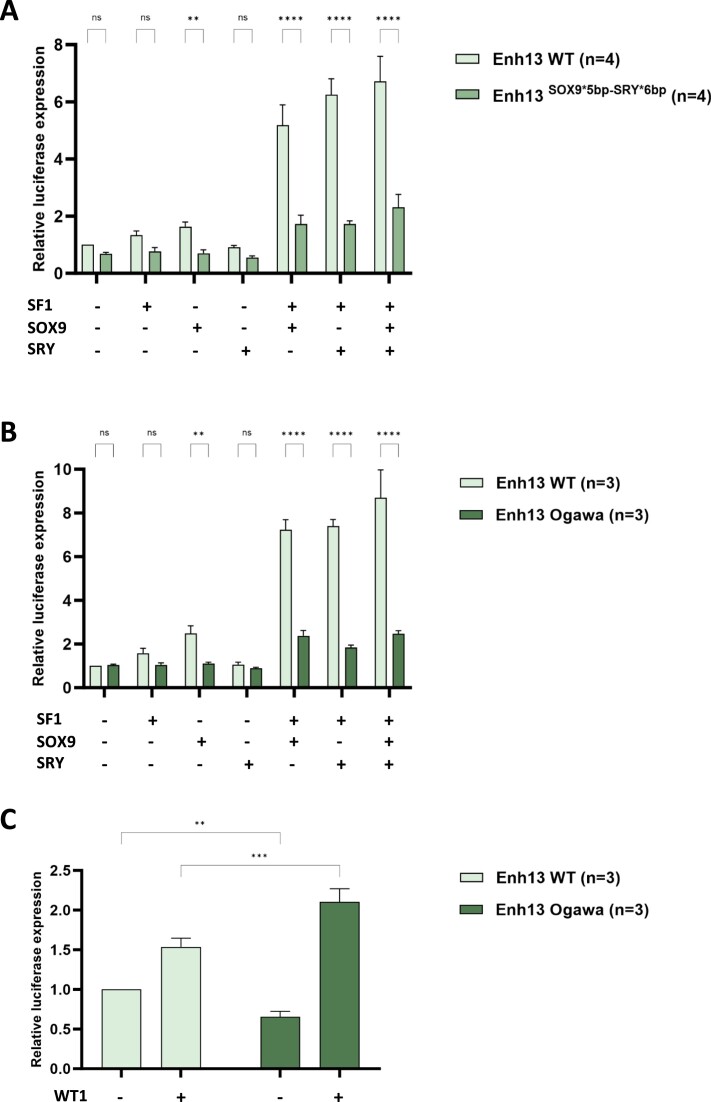
Luciferase Reporter Assays of the various mutations in TFBS in Enh13. (A–C) Luciferase reporter assays of HEK293T cells co-transfected with combinations expression plasmids of NR5A1, SOX9, SRY along with Luciferase construct containing the Enh13 WT, Enh13^SOX9*5bp^**^-^**^SRY*6bp^ (**A**) or Enh13 Ogawa containing the Poly G sequences (**B**). All values were normalized to the Enh13 WT luciferase activity with no expression plasmids added. Values were also normalized to the Renilla expression. (**C**) Luciferase reporter assays of HEK293T cells co-transfected with combinations expression plasmid of WT1 (-KTS) along with Luciferase construct containing the Enh13 WT and Enh13 Ogawa containing the Poly G sequences. All values were normalized to the Enh13 WT luciferase activity with no expression plasmids added. Means and SEM from at least three independent experiments are shown. Statistical analysis was done using two-way ANOVA followed by Tukey's/Dunnett's posttest. *P < 0.05, **P < 0.01, ***P < 0.001 and ****P < 0.0001, ns- not significant. WT, wild type, *n* = number of biological replicates.

To corroborate these *in vitro* EMSA data, we employed Luciferase enhancer reporter assay. To that aim, we cloned into the pGL4 Luciferase reporter vector either the *wild type* Enh13, the Enh13^SOX9*5bp^**^-^**^SRY*6bp^ (Figure 5A), or the Enh13 Ogawa sequence containing the Poly G sequences (Figure 5B). These vectors were transfected to HEK293T cells along with expression vectors of NR5A1, SOX9, SRY or combination of these. Analysis of the *wild type* Enh13 showed that addition of NR5A1, SOX9 or SRY alone only slightly activated enhancer activity, however, co-transfection with either NR5A1 and SOX9 or NR5A1 and SRY, or all 3 factors together significantly increased enhancer activity (Figure 5A, B). Interestingly, upon the use of the Enh13^SOX9*5bp^**^-^**^SRY*6bp^ vector (Figure 5A) or the Enh13 Ogawa vector (Figure 5B), a major decrease in enhancer activity is seen under all conditions, and mostly upon co-transfection of two or three factors together. These findings strongly support the *in vivo* mouse phenotypes and the EMSA results showing that both the microdeletions and Poly G substitutions destroy the binding capacity of SOX9 and SRY to their binding sites.

Next, we wanted to assess the effect of WT1 addition on enhancer activity of the *wild type* Enh13 compared to the Ogawa Enh13 (Figure 5C). A slight activation of Enh13 WT was seen in the presence of WT1, which can be explained by the presence of three WT1 BS present in mouse Enh13 as we described previously: one upstream of NR5A1 BS and two others downstream of SRY BS (15). Accordingly to our EMSA results, the presence of two additional WT1 BS in the Ogawa construct induced a significantly greater activation by the WT1 protein (Figure 5C).

Therefore, our data suggest that the addition of two WT1 BS in Enh13 of the Ogawa mutant recruits more WT1 protein on chromatin, possibly mediating partial rescue and hence a milder phenotype that does not result in sex reversal.

## Discussion

It is well established that both copy number variants (CNVs) as well as microdeletions/single nucleotide polymorphisms (SNPs) in genes involved in the sex determination path can lead to sex reversal in mice and DSD pathologies in humans (4–6). Yet, as many DSD patients fail to receive genetic diagnosis following WES, this suggest that many variants which lie in the non-coding parts in the genome are heavily involved. We and others have previously shown that CNV in Enh13 can lead to both 46,XY DSD and 46,XX DSD (15,25–27). Here we show that microdeletions in two TFBS in Enh13 can cause complete XY sex reversal. The fact that not only CNV, but also microdeletions in Enh13 can induce sex reversal implies that microdeletions /SNPs in gonad-specific enhancers may be a common cause to account for the many unexplained cases of DSD. This is likely to be true in a broader sense such that SNPs in other critical developmental enhancers may account for many other pathologies as well as cancer (1,52).

While individual BS mutations did not cause XY sex reversal, combined microdeletions in the SRY/SOX BS lead to complete sex reversal. This suggest that the two SRY/SOX BS are redundant and while the NR5A1 BS can slightly affect *Sox9* expression, it is not crucial. Interestingly, similar findings were shown at the regulation of the *Mis/Amh* promoter. Mice carrying homozygous NR5A1 BS mutations still expressed *Mis*, albeit at lower levels, while males homozygous for SOX9 BS were not able to induce *Mis* expression, resulting in persistence of Müllerian ducts in XY mutants (30,53). Interestingly, the SOX9 and more broadly, the SOXE subfamily, are known to mostly bind DNA as dimers via inverted repeats of the SOX consensus (51,54–56). The SOX9 binding site in Enh13 is a good half site, but it lacks the sequence of the second half site. Yet, EMSA experiments propose that both monomers and dimers can bind the SOX9 site in Enh13. This suggest that SOX9 can exert its regulatory effect even in the absence of the second half site. Moreover, a mutation that abrogates dimerization capability (A76E) of SOX9 has been described in a patient presenting with campomelic dysplasia but without sex reversal (56). This mutation affects the capacity of SOX9 to activate cartilage but not gonadal enhancers. Finally, the *Amh*promoter that is regulated by SOX9 contains only half SOX BS site (CTTTGAGA) and it mutation strongly abrogates *Amh* activation/expression by SOX9 *in vitro* (30) and *in vivo* (53). This suggest that SOX9 can exert its regulatory effect even in the absence of the second half site probably because of its interaction with other transcription factors as NR5A1 in the case of the *Amh* promoter (30).

Many studies on enhancer activity suggest that it is likely to be mediated by recruitment of specific transcription factors to the enhancer and promoter, which in turn induces physical proximity between the two, leading to activation of gene expression (33,57–59). Many genes, mostly developmental genes, are regulated by multiple, often redundant, regulatory elements (33). Hence, while we can relatively easily identify putative enhancers, enhancer redundancy poses a major challenge for exploring their mode of action in depth. The fact that *Sox9* expression in the testis, at the stages of sex determination, is regulated via a single crucial enhancer, deletion of which can lead to dramatic phenotype as sex reversal, suggest Enh13 as an attractive ‘model enhancer’ to explore questions related to enhancer function. Although the binding of TF is estimated to play a major role in enhancer activity, the precise mode of action and different mechanisms remain obscure (59). Some enhancers require cooperative binding of multiple TF to allow their function (60) while others rely on low-affinity, suboptimal, TFBS to precisely control gene expression (61–63). Alteration of these low affinity TFBS into consensus sites leads to aberrant gene expression and disease phenotypes (61–63). Our study shows *in vivo* that TFBS are the functional elements in enhancers and their targeted inactivation can lead to the same phenotype as deletion of the entire enhancer. The likelihood that one patient will contain two microdeletions in one enhancer are very low, but this study shows that alterations in TFBS within enhancers can mediate disease.

Several major challenges exist at investigating enhanceropathies and interpreting variants. The first is that the effect of the variant may be time- and tissue-specific and we rarely have access to these tissues at the relevant developmental time to assess the effect on gene expression (1). The current study provides causality between the variants, the effect on gene expression at the relevant tissue and developmental stage, and the resulting phenotype. A second challenge is to interpret how sequence variation at certain genomic location causes different clinical outcomes (1,52). Our study provides a remarkable *in vivo* example of that. In Ogawa *et al.* (41), the TFBS were destroyed by the insertion of poly G sequences, not creating any microdeletions hence leaving the overall length and genomic position of the enhancer intact. Yet, unintendedly, a novel TFBS was probably created. In our study, INDELs were introduced causing microdeletions in the TFBS. These microdeletions altered the overall length and genomic position of the enhancer. They could have also altered the distance between two nearby TFBS. It is fascinating to see how the two different mutations resulted in such different phenotypes. If other INDELS would have occurred, we could as well, unintendedly, create a novel BS. This suggest that caution should be taken when aiming to destroy a TFBS, and perhaps different and complementary approaches should be explored in parallel. From a clinical point of view, this *in vivo* comparison provides a molecular explanation to how individuals with different SNPs in the same enhancer may present with a range of phenotypes at different severities.

The sex determination process is highly dependent on timing and threshold levels of expression (14). For example, forced expression experiments of *Sry* in XX gonads, regulated by heat shock time-controlled promoters, have shown that if *Sry* starts to be expressed too early (before E11) or too late (after E11.25), testis development fails to initiate in XX, and an ovary will develop instead (64). Furthermore, mutant mice with reduced *Sry* expression produce ovaries or ovotestes. It is likely that in all of these cases, the delayed, or reduced, *Sry* levels do not cross the needed threshold to activate enough *Sox9* expression, and hence the testicular developmental path cannot be initiated and the ovarian path is initiated instead (65). Similar threshold sensitivity is seen with *Sox9* expression levels in the gonads. While in humans, loss of one allele of *SOX9* would lead to campomelic dysplasia, strongly associated with XY female development (20,21), in mice, male development occurs upon loss of one allele of *Sox9* in mixed genetic background (66) but leads to ovotestes formation in pure C57BL/6 genetic background (67). Indeed, we have previously shown that the threshold below which testis development will fail to occur is 25% of the normal *Sox9* levels in the mouse (68). Furthermore, when a weak *Nr5a1*-cre driver was used to delete *Sox9* in the gonads, only mild phenotypes appeared and not full XY sex reversal (22). This all suggest that if the reduction of *Sox9* expression in the gonads is not strong and pronounced, partial and weaker phenotypes appear. We hypothesize that this is the case with the poly G used in the Ogawa study (41) in which the poly G sequence enabled WT1 binding which rescued some of the expression of *Sox9*, leading to a mild phenotype.

## Supplementary Material

gkae178_Supplemental_File

## Data Availability

All data needed to evaluate the conclusions in the paper are present in the paper and/or the Supplementary Materials.
