# Supplementary material for: Two redundant transcription factor binding sites in a single enhancer are essential for mammalian sex determination
Source: Nucleic Acids Res. 2024 Mar 18;52(10):5514–28. doi: 10.1093/nar/gkae178 (PMC11162780; doi:10.1093/nar/gkae178)
Supplement: gkae178_Supplemental_File [file gkae178_supplemental_file.pdf]

Extended data for

Two redundant transcription factor binding sites in a single enhancer are essential for mammalian sex determination

Meshi Ridnik[1&], Elisheva Abberbock[1&], Veronica Alipov[1], Shelly Ziv Lhermann[1], Shoham Kaufman[1], Maor Lubman[1], Francis Poulat[2], Nitzan Gonen[1*]

*Corresponding author: Nitzan.Gonen@biu.ac.il

Extended data includes:

Figs. S1 to S10
Tables S1 to S7

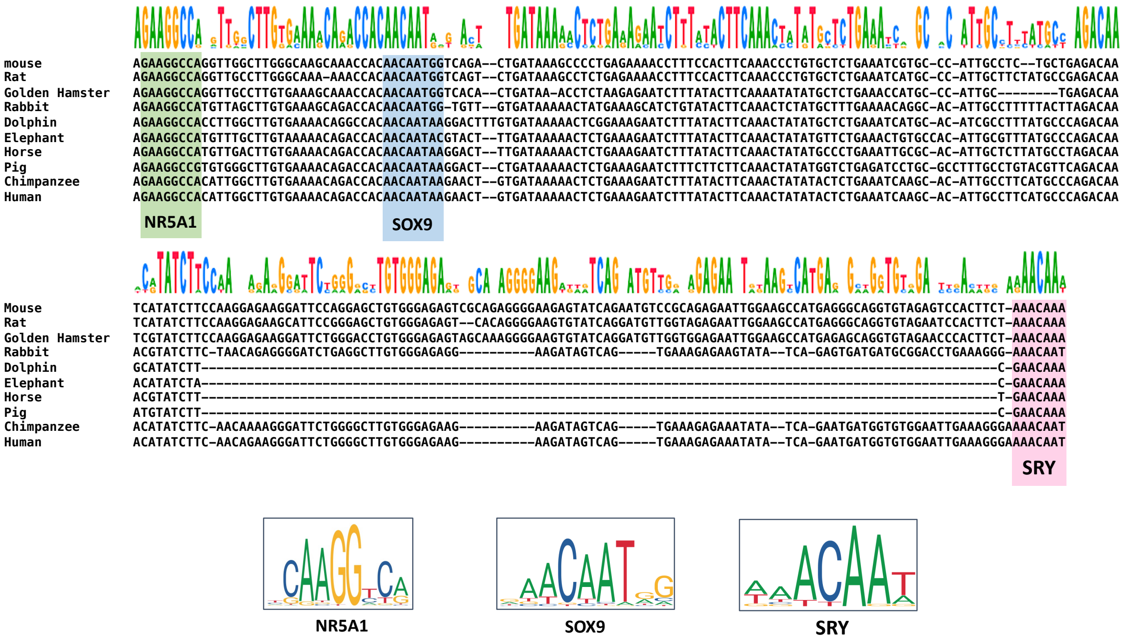

Supplementary Figure 1. Conservation analysis of Enh13 and locations of transcription factor binding motifs. The Enh13 sequence from 10 different mammals was compared using the Muscle tool from Snapgene. The Enh13 region between the NR5A1 to the SRY binding motifs is presented. The locations of the NR5A1 binding motif (Green), SOX9 binding motif (blue) and SRY binding motif (pink) are labelled. The consensus binding motifs of the NR5A1, SOX9 and SRY transcription factors are presented (based on JASPAR).

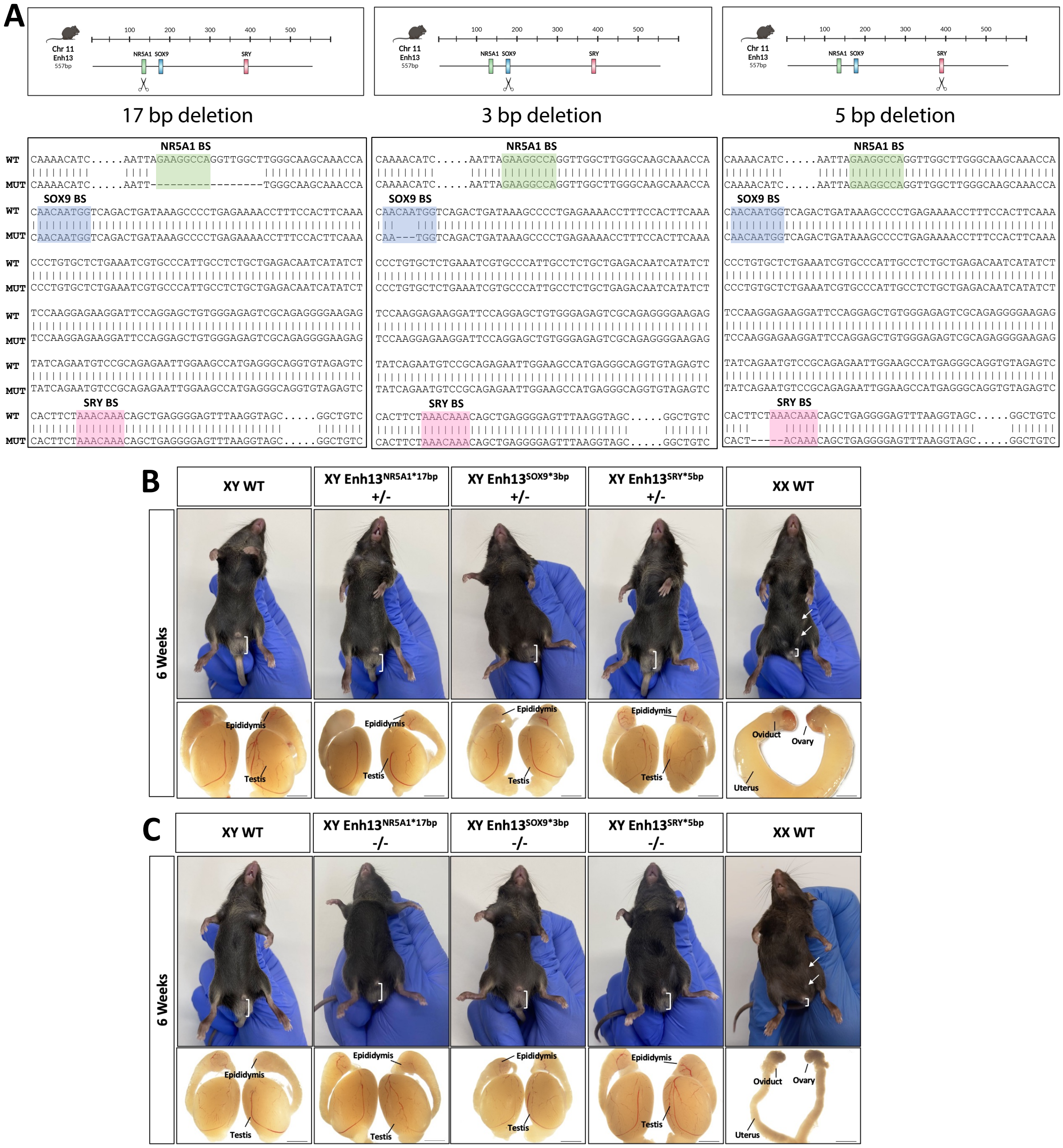

Supplementary Figure 2. Second independent mouse strains for individual TFBS mutations in Enh13. (A) Schematic representation of the CRISPR targeting approach and BLAST sequencing alignment between the WT Enh13 sequence (top) and homozygous mice mutated for Enh13 sequences (bottom) (NR5A1 BS- 17 bp deletion; Enh13^{NR5A1*17bp}, SOX9 BS- 3 bp deletion; Enh13^{SOX9*3bp}, SRY BS- 5 bp deletion; Enh13^{SRY*5bp}). (B-C) Bright field images of the external genitalia and gonads of 6 week-old mice of WT male (XY) and female (XX), and XY heterozygous (B) as well as homozygous (C) mice of the different mutations in individual TFBS in Enh13

(NR5A1 BS- 17 bp deletion; Enh13^{NR5A1*17bp}, SOX9 BS- 3 bp deletion; Enh13^{SOX9*3bp}, SRY BS- 5 bp deletion; Enh13^{SRY*5bp}). Scale bar represents 2000 µm. WT- *Wild Type*

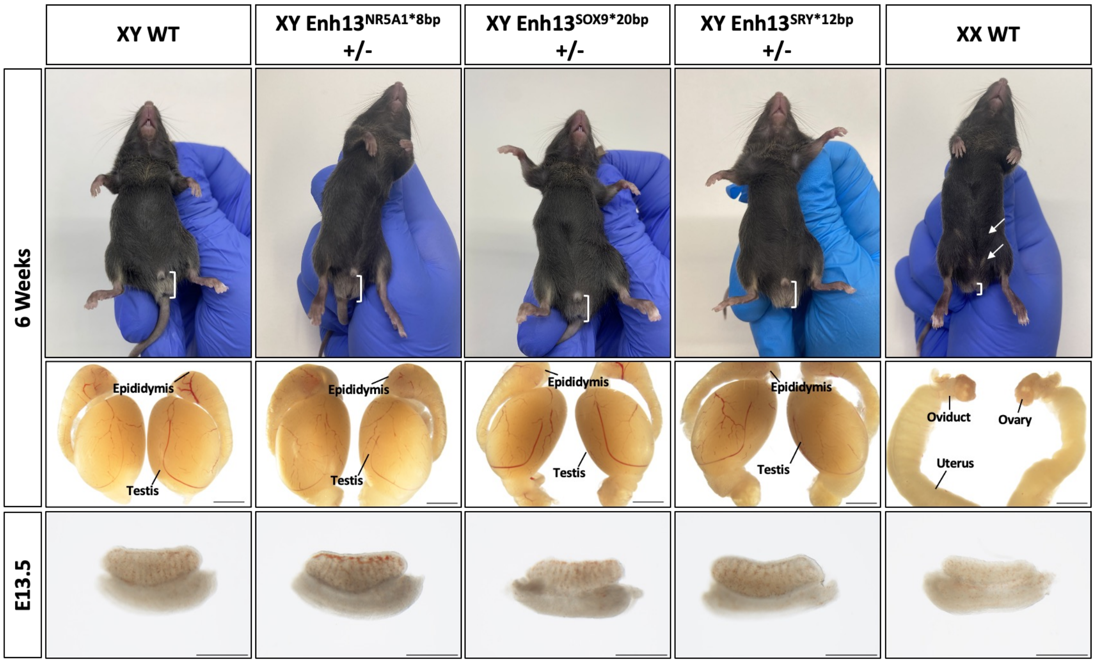

Supplementary Figure 3. Individual heterozygous mutations in TFBS of Enh13 do not affect sex appearance. Bright field images of the external genitalia and gonads of 6 week-old mice and E13.5 embryos of WT male (XY) and female (XX), and XY heterozygous mice of the different mutations in individual TFBS (NR5A1 BS- 8 bp deletion; $Enh13^{NR5A1*8bp}$, SOX9 BS- 20 bp deletion; $Enh13^{SOX9*20bp}$, SRY BS- 12 bp deletion; $Enh13^{SRY*12bp}$). Scale bar represents 2000 µm for 6 week-old gonads and 500 µm for E13.5 gonads. WT- *Wild Type*

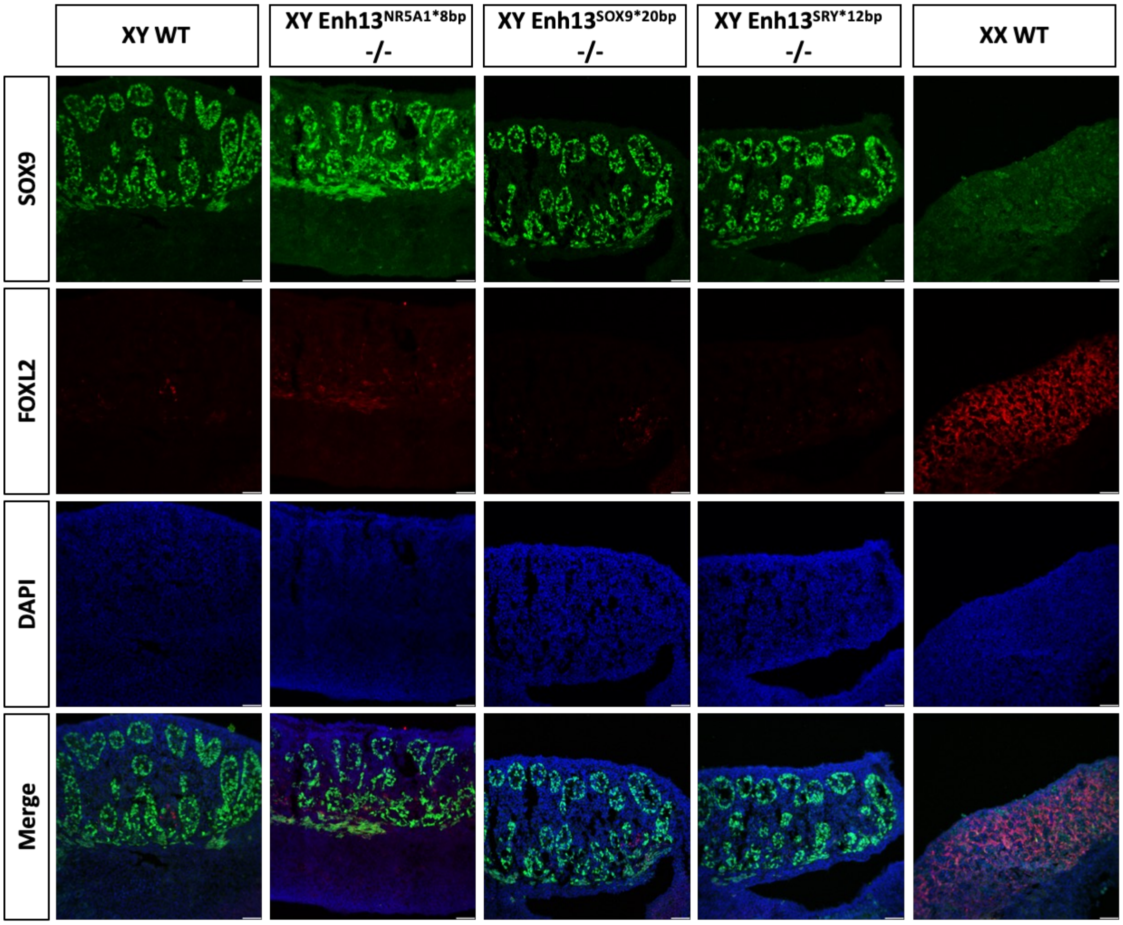

Supplementary Figure 4. Immunostaining of E13.5 gonads from embryos carrying individual TFBS mutations in Enh13. Immunostaining of E13.5 gonads from WT XY and XX as well as XY homozygous embryos of the different mutations in individual TFBS in Enh13 (NR5A1 BS- 8 bp deletion; $Enh13^{NR5A1*8bp}$, SOX9 BS- 20 bp deletion; $Enh13^{SOX9*20bp}$, SRY BS- 12 bp deletion; $Enh13^{SRY*12bp}$). Gonads were stained for Sertoli-marker SOX9 (green), Granulosa-marker FOXL2 (red) and DAPI (blue). Scale bars represent 50 µm. WT- *Wild Type*.

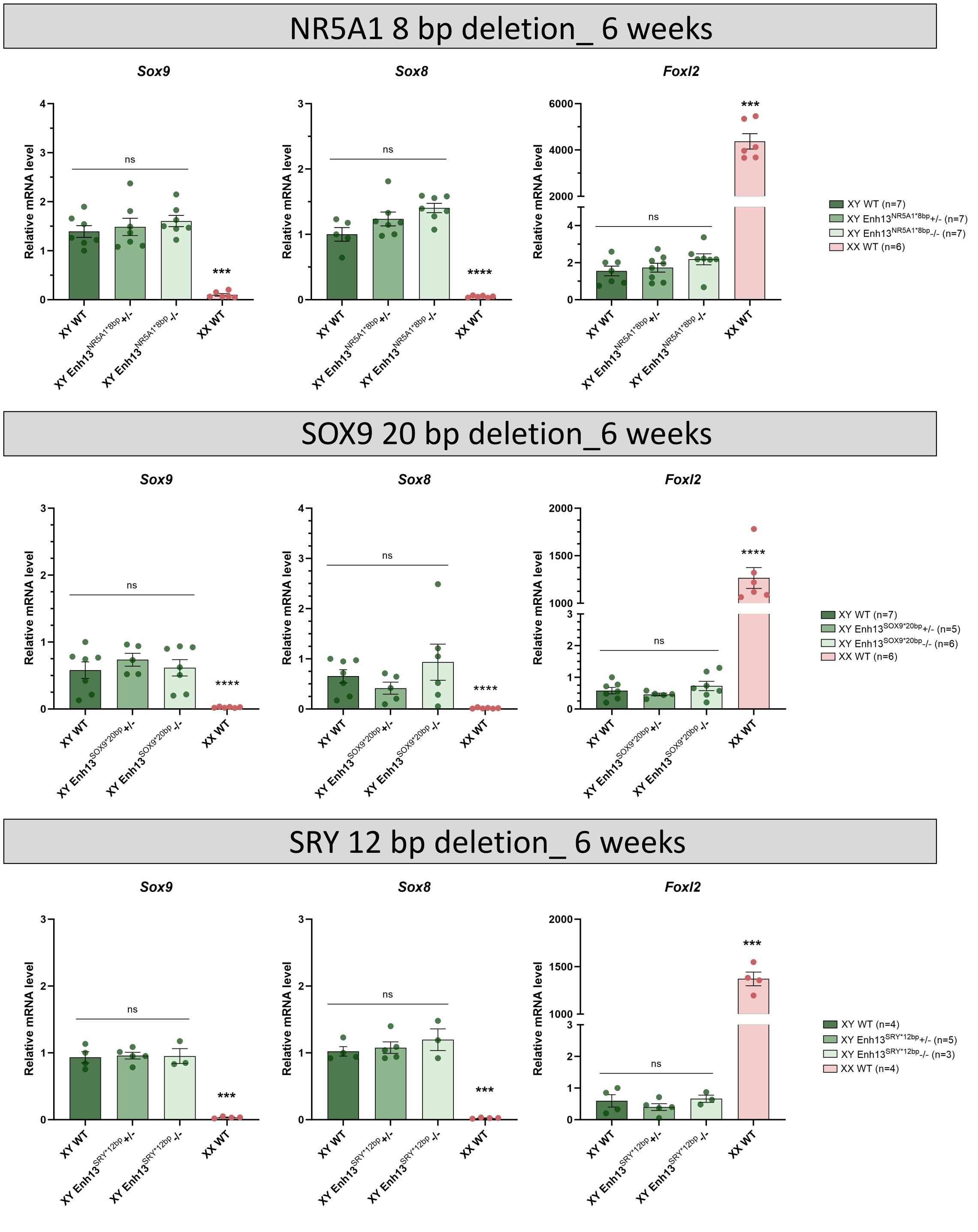

Supplementary Figure 5. Gene expression analysis of 6 week-old gonads of mice carrying individual TFBS mutations in Enh13. Real-time quantitative PCR analysis of genes involved male (*Sox9* and *Sox8*) and in female (*Foxl2*) gonadal sex determination at 6 week-old gonads of mice carrying the different mutations in individual TFBS in Enh13 (NR5A1 BS- 8 bp deletion; Enh13^{NR5A1*8bp}, SOX9 BS- 20 bp deletion; Enh13^{SOX9*20bp}, SRY BS- 12 bp deletion;

Enh13^{SRY*12bp}). Data are presented as mean $2^{-\Delta\Delta Ct}$ values ±SEM, normalized to the housekeeping gene *Hprt*. Sample size indicated next to each genotype represents the number of individuals harvested (n). Statistical analysis was done using one-way ANOVA followed by Dennett's posttest. $*P < 0.05$, $**P < 0.01$, $***P < 0.001$, and $****P < 0.0001$, ns- not significant. WT- *Wild Type*.

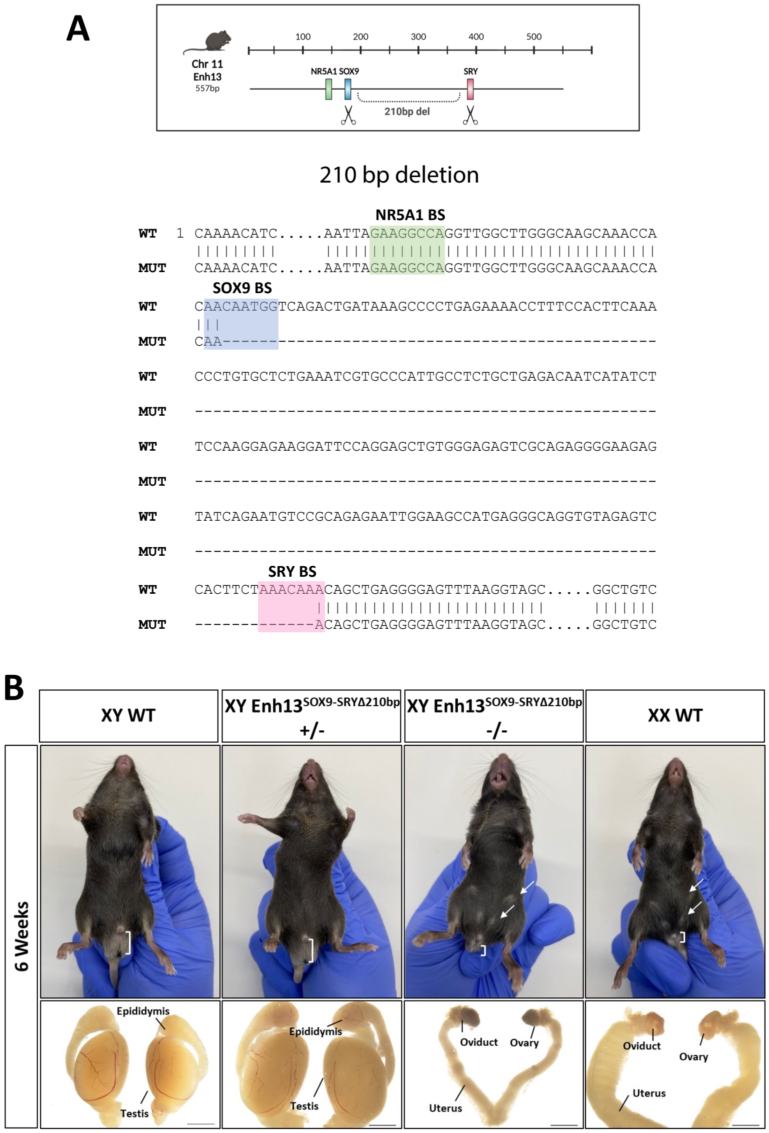

Supplementary Figure 6. A 210 bp deletion removing the SOX9 and SRY BS in a second independent mouse strain leads to complete XY sex reversal. (A) Schematic representation of the CRISPR targeting approach and BLAST sequencing alignment between the WT Enh13 sequence (top) and homozygous mice carrying a 210 bp deletion within Enh13 deleting the SOX9 and SRY TFBS and the sequence between them (Enh13$^{SOX9\text{-}SRY\Delta 210bp}$) (B) Bright field images of the external genitalia and gonads of 6 week-old mice of WT male (XY) and female (XX) mice, and the XY heterozygous and homozygous mice carrying the 210 bp deletion between the SOX9 and SRY TFBS ((Enh13$^{SOX9\text{-}SRY\Delta 210bp}$). Scale bar represents 2000 µm. WT- *Wild Type*

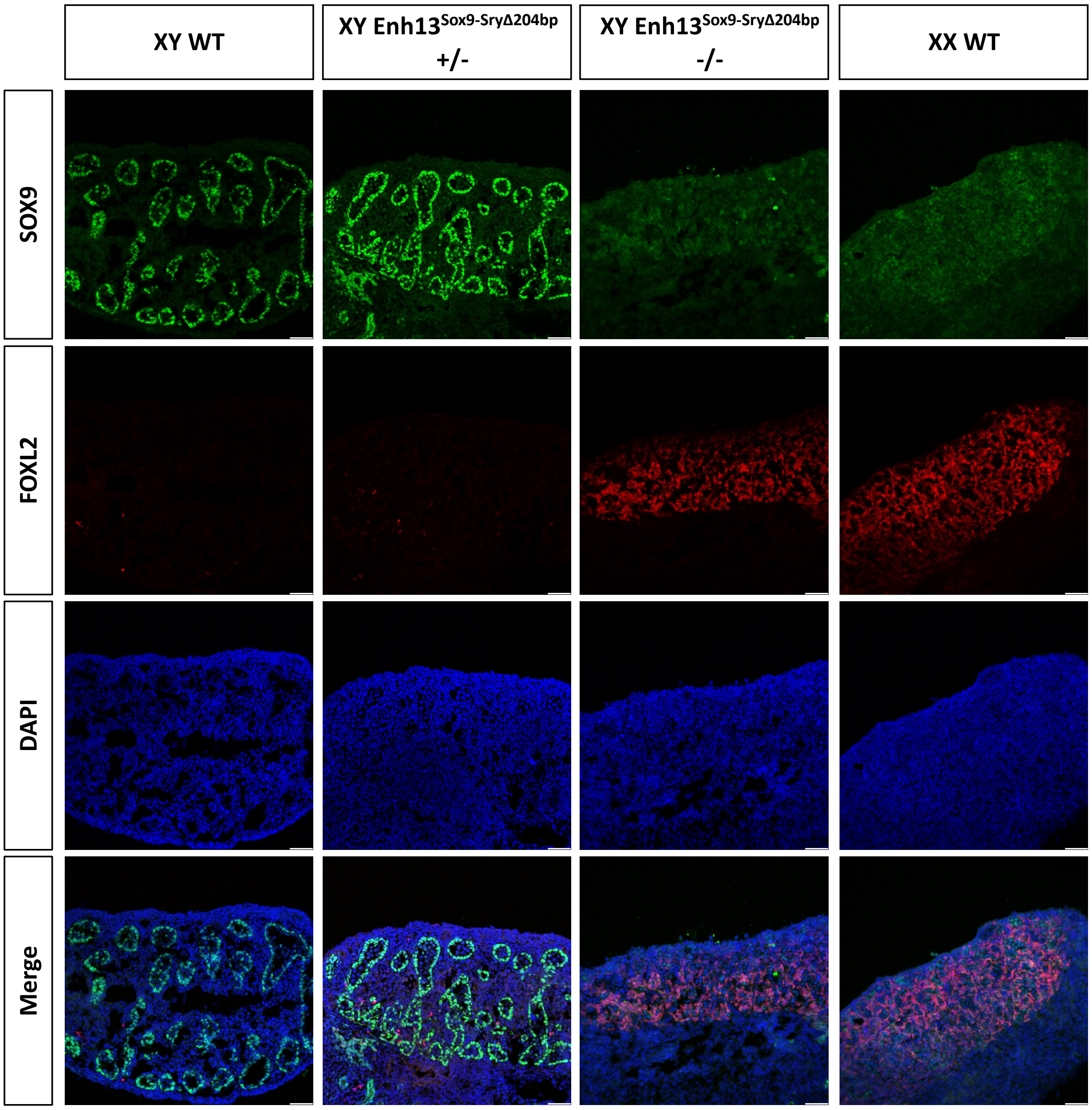

Supplementary Figure 7. A 204 bp deletion within Enh13, removing the SOX9 and SRY BS leads to XY sex reversal in E13.5 embryos. Immunostaining of E13.5 gonads from WT XY and XX as well as XY heterozygous and homozygous embryos carrying a 204 bp deletion within Enh13 removing the SOX9 and SRY TFBS ($Enh13^{SOX9\text{-}SRY\Delta 204bp}$). Gonads were stained for Sertoli-marker SOX9 (green), granulosa-marker FOXL2 (red) and DAPI (blue). Scale bars represent 50 μm. WT- *Wild Type*

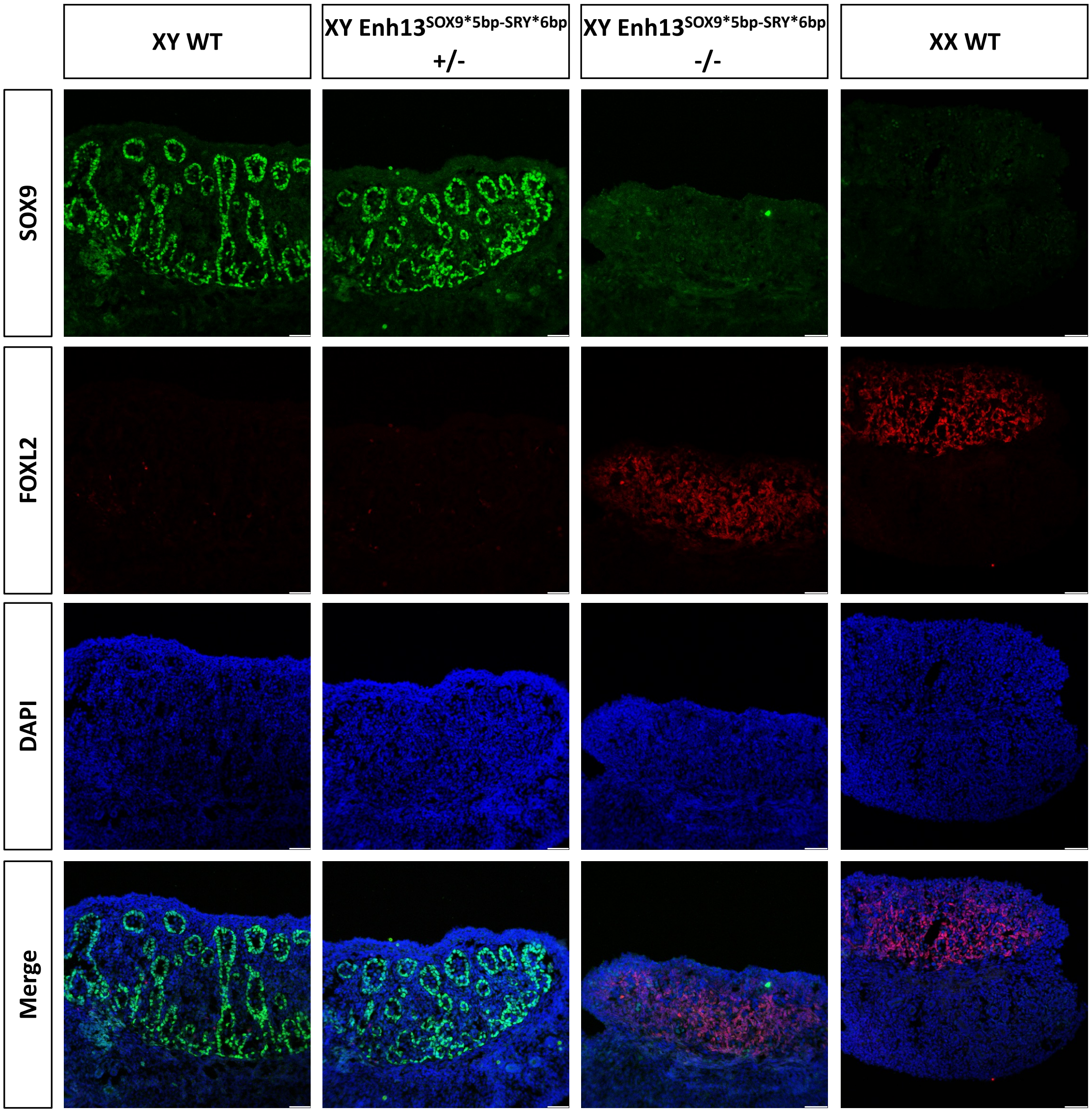

Supplementary Figure 8. Combined mutation of the SOX9 and SRY TFBS leads to XY sex reversal in E13.5 embryos. Immunostaining of E13.5 gonads from WT XY and XX as well as XY heterozygous and homozygous embryos carrying combined mutations of the SOX9 and SRY TFBS of Enh13 ($Enh13^{SOX9*5bp-SRY*6bp}$). Gonads were stained for Sertoli-marker SOX9 (green), granulosa-marker FOXL2 (red) and DAPI (blue). Scale bars represent 50 µm. WT- *Wild Type*

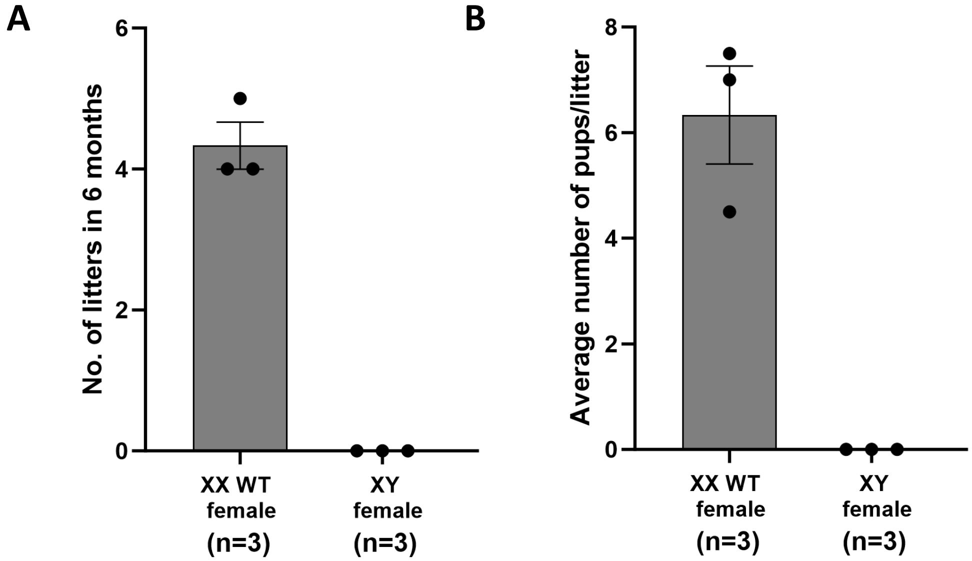

Supplementary Figure 9. Fertility tests of XX wild type females and XY Enh13$^{SOX9*5bp\text{-}SRY*6bp}$ females. (A) Number of litters born per female when mated with C57BL/6J male for a period of 6 months from either XX WT females or XY Enh13$^{SOX9*5bp\text{-}SRY*6bp}$ females. n= number of females recorded, biological replicates. (B) Average number of pups born per litter from either XX WT females or XY Enh13$^{SOX9*5bp\text{-}SRY*6bp}$ females mated with C57BL/6J male for a period of 6 months. n= number of females recorded, biological replicates.

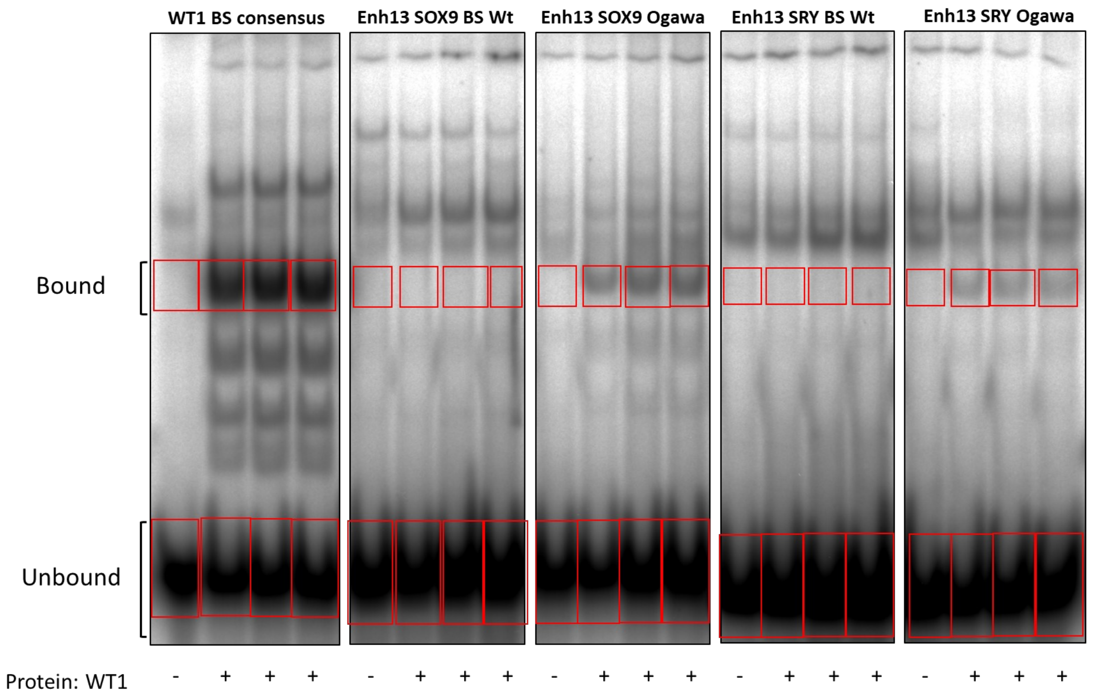

Supplementary Figure 10. EMSA analysis of the binding capacity of WT1 to various sequences of Enh13. EMSA using probes containing either a WT1 consensus sequence (left), or two versions of probes for the Enh13 SOX9 BS (two middle gels) or the Enh13 SRY BS (two right gels). The first probe each pair contains is the wild type Enh13 sequence and the second probe is a probe containing a Poly G sequence to substitute the TFBS as used by Ogawa et al. All five probes were loaded in the absence or presence of the WT1 protein. The top red box labels that WT1-bound probe, and the bottom red box labels the unbound probe. For each probe, three biological repeats were loaded. Figure 4G presents the quantification of this analysis. *Wt- Wild Type,* WT1- Wilms' tumor 1

Table S1. Primers used for genotyping mice and cloning

Target	Primer name	Description	Sequence 5' to 3'	Product and size
Sex	Sex_F	X/Y chromosome	5'-GATGATTTGAGTGGAAATGTGAGGTA-3'	Sex_F + Sex_R: 280bp in XY; 685bp,660bp and 480bp in XX (McFarlane et al., 2013)
	Sex_R	X/Y chromosome	5'-CTTATGTTTATAGGCATGCACCATGTA-3'	
Enh13 616bp	Enh13_F3	Flanks 5' end of Enh13	5'-TTAAGAAGGTAGACCCAGAG-3'	Enh13_F3 + Enh13_R4: 616bp in WT allele or INDELs; 412bp in deleted alleles (204 or 210 bp del)
	Enh13_R4	Flanks 3' end of Enh13	5'-CCTCATTCTTTGCAGAAAGC-3'	
NR5A1 BS 8bp del	Enh13_NR5A1_WT_F2	Flanks 5' end of WT NR5A1 BS	5'-TGAGGAATTAGAAGGCCAG-3'	NR5A1_WT_F2 + Enh13_R5: 464bp in WT allele
	Enh13_NR5A1_8bp_del_F2	Flanks 5' end of mutated NR5A1 BS	5'-AGCTGAGGAATCAGGTTG-3'	NR5A1_8bp_del_F2 + Enh13_R5: 454bp in mutated allele
	Enh13_R5	Flanks 3' end of Enh13	5'-CATATCACTCAGCTCAGACA-3'	
Sox9 BS 20bp del	Sox9_20bp_WT_F2	Flanks 5' end of WT SOX9 BS	5'-GCAAGCAAACCACAACAAT-3'	Sox9_20bp_WT_F2 + Sox9_20bp_WT_R1: 280bp in WT allele
	Sox9_20bp_mut_F2	Flanks 5' end of mutated SOX9 BS	5'-GGTTGGCTTGGGCAAGAC-3'	Sox9_20bp_mut_F2 + Enh13_R4: 292bp in mutated allele
	Sox9_20bp_WT_R1	Flanks 3' end within Enh13	5'-GGCTGATTAGAACACACTG-3'	
	Enh13_R4	Flanks 3' end of Enh13	5'-CCTCATTCTTTGCAGAAAGC-3'	
SRY BS 12bp del	Enh13_F7	Flanks 5' end of WT SRY BS	5'-GAGTCCACTTCTAAACAAAC-3'	Enh13_F7 + Enh13_R5: 228bp in WT allele
	Enh13_F7_12bp_del	Flanks 5' end of mutated SRY BS	5'-TAGAGTCCACAGCTGAG-3'	Enh13_F7_12bp_del + Enh13_R5: 218bp in mutated allele
	Enh13_R5	Flanks 3' end of Enh13	5'-CATATCACTCAGCTCAGACA-3'	
NR5A1 BS 17bp del	NR5A1_17bp_WT_F1	Flanks 5' end of WT NR5A1 BS	5'-GAGGAATTAGAAGGCCAGG-3'	SF1_17bp_WT_F1 + SF1_17bp_R1: 263bp in WT allele
	NR5A1_17bp_mut_F2	Flanks 5' end of mutated NR5A1 BS	5'-AGGAATGTGGGCAAGCAA-3'	SF1_17bp_mut_F2 + SF1_17bp_R1: 245bp in mutated allele
	NR5A1_17bp_R1	Flanks 3' end within Enh13	5'-CCTCAGCTGTTTGTTTAGAAG-3'	
Sox9 BS 3bp del	Sox9_3bp_WT_F1	Flanks 5' end of WT SOX9 BS	5'-CAACAATGGTCAGACTGAT-3'	Sox9_3bp_WT_F1 + Sox9_3bp_WT_R1: 268 bp in WT allele
	Sox9_3bp_mut_F1	Flanks 5' end of mutated SOX9 BS	5'-CACAATGGTCAGACTGATA-3'	Sox9_3bp_mut_F1 + Enh13_R5: 420bp in mutated allele
	Sox9_3bp_WT_R1	Flanks 3' end within Enh13	5'-GGCTGATTAGAACACACTG-3'	
	Enh13_R5	Flanks 3' end of Enh13	5'-CATATCACTCAGCTCAGACA-3'	
SRY BS 5 del	Enh13_F7	Flanks 5' end of WT SRY BS	5'-GAGTCCACTTCTAAACAAAC-3'	F7 + R5: 228bp in WT allele
	Enh13_F7_5bp_del	Flanks 5' end of mutated SRY BS	5'-AGTCCACTACAAACAGCT-3'	F7_5bp_del + R5: 222bp in mutated allele
	Enh13_R5	Flanks 3' end of Enh13	5'-CATATCACTCAGCTCAGACA-3'	
Enh13 557bp	Enh13_BglII_F	Flanks 5' end of Enh13 and adds BglII site	5'-CAGAGATCTCAAAACATCCAGGTGGG-3'	Amplified 557bp of Enh13 while adding restriction enzyme sites for BglII and HindIII
	Enh13_HindIII_R	Flanks 3' end of Enh13 and adds HindIII site	5'-CTCAAGCTTGACAGCCACATGCAATC-3'	
PGL4.26 MCS	RV3	Sits upstream to the MCS in PGL4.26	5'CTAGCAAAATAGGCTGTCCC-3'	Primer used for sequencing the PGL4.26 plasmid

Table S2. sgRNA used for CRISPR genome editing

Application	sgRNA name	Sequence 5' to 3'	PAM
Enh13_SF1_BS sgRNA	SF1_BS_G1	AGCTGAGGAATTAGAAGGCC	AGG
Enh13_SOX9_BS sgRNA	SOX9_BS_G1	TATCAGTCTGACCATTGTTG	TGG
Enh13_SRY_BS sgRNA	SRY_BS_G1	CTCAGCTGTTTGTTTAGAAG	TGG

Table S3. Antibodies/ Dyes used in this study

Target	Host	Manufacture	Cat. number	Dilution	Antibody type	Used for staining	Cell type
αSOX9	Rabbit	Millipore	AB5535	1:2000	Primary	Adult gonads (6W)	Sertoli cells
αFOXL2	Goat	Novus	NB100-1277	1:250	Primary	Adult gonads (6W)	Granulosa cells
αDDX4	Mouse	Abcam	ab27591	1:300 for **males** /<br>1:2000 for **females**	Primary	Adult gonads (6W)	Germ cells
αSOX9	Goat	R&D systems	AF3075	1:300	Primary	Embryonic gonads (E13.5)	Sertoli cells
αFOXL2	Rabbit	Abcam	ab246511	1:300	Primary	Embryonic gonads (E13.5)	Granulosa cells
αGCNA1 (TRA98)	Rat	Abcam	ab82527	1:200	Primary	Embryonic gonads (E13.5)	Germ cells
αRabbit-Alexa flour 488	Donkey	Invitrogen	A-21206	1:500	Secondary	Adult gonads (6W)	-
αGoat-Alexa flour 568	Donkey	Invitrogen	A-11057	1:500	Secondary	Adult gonads (6W)	-
αMouse-Alexa flour 647	Donkey	Invitrogen	ab150111	1:500	Secondary	Adult gonads (6W)	-
αGoat-Alexa flour 488	Donkey	Invitrogen	A-11055	1:500	Secondary	Embryonic gonads (E13.5)	-
αRabbit-Alexa flour 568	Donkey	Invitrogen	A-10042	1:500	Secondary	Embryonic gonads (E13.5)	-
αRat-Alexa flour 647	Donkey	Abcam	ab150155	1:500	Secondary	Embryonic gonads (E13.5)	-
DAPI	-	Invitrogen	D-1306	300nM	Dye	All	Nucleic acid

Table S4. Primers used for quantitative RT-PCR			
**Primer name**	**Gene**	**Marker of**	**Sequence 5' to 3'**
Sox9 F	*Sox9*	Sertoli cells	AAGAAAGACCACCCCGATTACA
Sox9 R			CAGCGCCTTGAAGATAGCATT
Sox8 F	*Sox8*	Sertoli cells	AGCGAGAAGAGGCCGTTTG
Sox8 R			TCAGTACCAGAGTCTGAGTCG
Foxl2 F	*Foxl2*	Granulosa cells	CGGCATCTACCAGTACATCATAGC
Foxl2 R			GCACTCGTTGAGGCTGAGGTTG
Hprt F	*Hprt*	House keeping gene	GCTTGCTGGTGAAAAGGACCTCTCGAAG
Hprt R			CCCTGAAGTACTCATTATAGTCAAGGGCAT

Table S5. Probes used for EMSA		
**Probe name and target site**		**Sequence**
NR5A1 BS	Enh13 Wt	CTGAGGAATTA**GAAGGCCA**GGTTGGC
	Ridnik et al.	CTGAGGAAT--------CAGGTTGGC
SOX9 BS	Enh13 Wt	GGCTTGGGCAAGCAAACCAC**AACAATGG**TCAGACTGATAA
	Ridnik et al.	GGCTTGGGCAA--------------------GACTGATAA
	Ogawa et al.	GGCTTGGGCAAGCAAACCA**GGGGGGGGG**TCAGACTGATAA
SRY BS	Enh13 Wt	GTGTAGAGTCCACTTCT**AAACAAA**CAGCTGAGGGG
	Ridnik et al.	GTGTAGAGTCC------------**A**CAGCTGAGGGG
	Ogawa et al.	GTGTAGAGTCCACT**GGGGGGGGGA**CAGCTGAGGGG
WT1 BS	Consensus	GGCTTGGGCAAGC**GGGGGAGG**ACAATGGTCAGACTGATAA
Binding motifs are labelled in red, deletions are labelled with '-'		

Table S6. EMSA measurements quantification						
**Well**	**Probe**	**Lysate**	**Bound**	**WT1 Bound (Bound WT1-C)**	**Unbound (free probe)**	**% of bound**
1	WT1 BS Consensus	Control (-WT1)	4407.5		270257.0	
2	WT1 BS Consensus	+WT1	49756.2	45348.7	286733.5	15.82
3	WT1 BS Consensus	+WT1	53449.7	49042.2	298587.6	16.42
4	WT1 BS Consensus	+WT1	53794.3	49386.8	302310.2	16.34
5	Enh13 SOX9 BS Wt	Control (-WT1)	4746.1		310524.5	
6	Enh13 SOX9 BS Wt	+WT1	7167.3	2421.2	310999.8	0.78
7	Enh13 SOX9 BS Wt	+WT1	7199.5	2453.4	316824.5	0.77
8	Enh13 SOX9 BS Wt	+WT1	7923.9	3177.8	320281.9	0.99
9	Enh13 SOX9 Ogawa	Control (-WT1)	1622.2		304856.8	
10	Enh13 SOX9 Ogawa	+WT1	22858.3	21236.1	297455.4	7.14
11	Enh13 SOX9 Ogawa	+WT1	30621.2	28999.0	309463.6	9.37
12	Enh13 SOX9 Ogawa	+WT1	32063.1	30441.0	315056.2	9.66
13	Enh13 SRY BS Wt	Control (-WT1)	3732.1		310682.5	
14	Enh13 SRY BS Wt	+WT1	5581.0	1848.9	319770.6	0.58
15	Enh13 SRY BS Wt	+WT1	5533.3	1801.3	323823.9	0.56
16	Enh13 SRY BS Wt	+WT1	5469.8	1737.7	324031.4	0.54
17	Enh13 SRY Ogawa	Control (-WT1)	4808.8		319115.1	
18	Enh13 SRY BS Wt	+WT1	15483.5	10674.7	312833.8	3.41
19	Enh13 SRY BS Wt	+WT1	16625.1	11816.2	311020.9	3.80
20	Enh13 SRY BS Wt	+WT1	17315.3	12506.5	308961.0	4.05

Table S7. Ordinary one-way ANOVA with multiple comparisons- EMSA experiments				
Tukey's multiple comparisons test	Mean Diff	95,00% CI of diff	Below threshold?	Summary
WT1 BS vs. *SOX9 Wt*	15.34	13,59 to 17,10	Yes	****
WT1 BS vs. SOX9 Ogawa *et al*	7.468	5,714 to 9,223	Yes	****
WT1 BS vs. SRY *Wt*	15.64	13,88 to 17,39	Yes	****
WT1 BS vs. SRY Ogawa *et al*	12.44	10,68 to 14,19	Yes	****
SOX9 Wt vs. SOX9 Ogawa *et al*	-7.876	-9,630 to -6,121	Yes	****
SOX9 Wt vs. SRY *Wt*	0.2914	-1,463 to 2,046	No	ns
SOX9 Wt vs. SRY Ogawa *et al*	-2.905	-4,659 to -1,150	Yes	**
SOX9 Ogawa *et al* vs. SRY *Wt*	8.167	6,413 to 9,922	Yes	****
SOX9 Ogawa *et al* vs. SRY Ogawa *et al*	4.971	3,216 to 6,725	Yes	****
SRY *Wt* vs. SRY Ogawa *et al*	-3.196	-4,951 to -1,442	Yes	***
Test details	Mean 1	Mean 2	Mean Diff	SE of diff
WT1 BS vs. *SOX9 Wt*	16.19	0.8484	15.34	0.5331
WT1 BS vs. SOX9 Ogawa *et al*	16.19	8.724	7.468	0.5331
WT1 BS vs. SRY *Wt*	16.19	0.5569	15.64	0.5331
WT1 BS vs. SRY Ogawa *et al*	16.19	3.753	12.44	0.5331
SOX9 Wt vs. SOX9 Ogawa *et al*	0.8484	8.724	-7.876	0.5331
SOX9 Wt vs. SRY *Wt*	0.8484	0.5569	0.2914	0.5331
SOX9 Wt vs. SRY Ogawa *et al*	0.8484	3.753	-2.905	0.5331
SOX9 Ogawa *et al* vs. SRY *Wt*	8.724	0.5569	8.167	0.5331
SOX9 Ogawa *et al* vs. SRY Ogawa *et al*	8.724	3.753	4.971	0.5331
SRY *Wt* vs. SRY Ogawa *et al*	0.5569	3.753	-3.196	0.5331

****: < 0.0001
***:<0.0002
**: <0.0332
